# Vascular senescence and leak are features of the early breakdown of the blood–brain barrier in Alzheimer’s disease models

**DOI:** 10.1007/s11357-023-00927-x

**Published:** 2023-10-02

**Authors:** Ka Ka Ting, Paul Coleman, Hani Jieun Kim, Yang Zhao, Jocelyne Mulangala, Ngan Ching Cheng, Wan Li, Dilini Gunatilake, Daniel M. Johnstone, Lipin Loo, G. Gregory Neely, Pengyi Yang, Jürgen Götz, Mathew A. Vadas, Jennifer R. Gamble

**Affiliations:** 1 Vascular Biology Program, Centenary Institute, Camperdown, NSW Australia; 2 Computational Systems Biology Group, Children’s Medical Research Institute, Faculty of Medicine and Health, The University of Sydney, Westmead, NSW 2145 Australia; 3 School of Medicine & Holistic Integrative Medicine, Nanjing University of Chinese Medicine, Nanjing, 210023 Jiangsu China; 4 Department of General Surgery, Jiangsu Province Hospital of Chinese Medicine, Affiliated Hospital of Nanjing University of Chinese Medicine, Nanjing, 210029 China; 5 School of Medical Sciences, University of Sydney, Camperdown, NSW Australia; 6 School of Biomedical Sciences & Pharmacy, University of Newcastle, Callaghan, NSW Australia; 7 Charles Perkins Centre, Dr. John and Anne Chong Lab for Functional Genomics, Centenary Institute, & School of Life and Environmental Sciences, University of Sydney, Camperdown, NSW Australia; 8 Clem Jones Centre for Ageing Dementia Research, Queensland Brain Institute, The University of Queensland, Brisbane, Australia; 9 Heart Research Institute, Sydney, NSW, Australia

**Keywords:** Alzheimer’s disease, Blood–brain barrier, Senescence, Vascular leak, VE-cadherin, Pericytes

## Abstract

Alzheimer’s disease (AD) is an age-related disease, with loss of integrity of the blood–brain barrier (BBB) being an early feature. Cellular senescence is one of the reported nine hallmarks of aging. Here, we show for the first time the presence of senescent cells in the vasculature in AD patients and mouse models of AD. Senescent endothelial cells and pericytes are present in APP/PS1 transgenic mice but not in wild-type littermates at the time of amyloid deposition. In vitro, senescent endothelial cells display altered VE-cadherin expression and loss of cell junction formation and increased permeability. Consistent with this, senescent endothelial cells in APP/PS1 mice are present at areas of vascular leak that have decreased claudin-5 and VE-cadherin expression confirming BBB breakdown. Furthermore, single cell sequencing of endothelial cells from APP/PS1 transgenic mice confirms that adhesion molecule pathways are among the most highly altered pathways in these cells. At the pre-plaque stage, the vasculature shows significant signs of breakdown, with a general loss of VE-cadherin, leakage within the microcirculation, and obvious pericyte perturbation. Although senescent vascular cells were not directly observed at sites of vascular leak, senescent cells were close to the leak area. Thus, we would suggest in AD that there is a progressive induction of senescence in constituents of the neurovascular unit contributing to an increasing loss of vascular integrity. Targeting the vasculature early in AD, either with senolytics or with drugs that improve the integrity of the BBB may be valid therapeutic strategies.

**Supplementary information:**

The online version contains supplementary material available at 10.1007/s11357-023-00927-x.

## Introduction

Alzheimer’s disease (AD) is an age-related neurodegenerative brain disease having the hallmarks of extracellular amyloid-β (Aβ) plaques, intracellular neurofibrillary tangles and neuroinflammation, and neuronal and synaptic loss [1, 2]. The failure of clinical trials of Aβ-clearing therapeutics to impact on AD progression and cognitive outcome has called into question the amyloid hypothesis as central for AD [3] or that delivery of these therapies have been too late in the disease progression or at efficacious concentrations [4]. However, the evidence that AD is primarily a vascular disease is strong. Changes in the neurovascular unit or blood–brain barrier (BBB) have been reported as an early feature [5, 6] as well as significant and widespread vascular leak [7]. Furthermore, risk factors, such as hypertension, diabetes, and hypercholesterolemia for dementia and that for cerebrovascular disease, overlap [7–10]. Strengthening this concept, APOE4, the major genetic risk factor for AD, impairs the BBB [9] and cerebrovascular integrity [10]. Furthermore, the micro-haemorrhagic bleeds in aged or damaged vasculature are surrounded by and associated with plaque-like pathology supporting the possibility that one of the functions of Aβ is to “plug” the bleeds and the plaques to sequester toxic products into an insoluble “scab” [11–16]. Indeed, both human and murine transgenic studies have shown that the breakdown of the BBB can lead to accumulation of blood-derived proteins such as albumin, fibrinogen, and iron [17, 18] and the chronic leakage of these proteins is associated with progressive neurodegeneration and cognitive impairment [19].

The BBB is primarily composed of brain endothelial cells (ECs), astrocytes end-feet, and pericytes [19]. Furthermore, the perivascular space of the BBB at least at large blood vessels and venules is occupied by perivascular macrophages [20]. The BBB is selective and semi-permeable, restricting the free diffusion of molecules between the central nervous system (CNS) and the peripheral circulation [17]. This selective nature is mediated through two major components of the ECs; influx/efflux transporters and cell–cell adhesion structures, together inhibiting transcellular and paracellular diffusion, respectively [21]. Additional to these properties, the brain endothelium has metabolic enzymes that contribute to a protective barrier against neurotransmitters, drug, and toxins (review by [22]). Blood vessel integrity depends on a continuous layer of ECs. The intimate cell–cell contact of ECs is ensured by a set of adhesion molecules localized to cell junctions. The main adhesion structures are the tight junctions (TJs) and adherens junctions (AJs). Claudin-5 is a key TJ protein that is highly enriched in brain ECs [23–25] and its dynamic changes play a major role in the paracellular movement of molecules [26, 27]. Furthermore, it can regulate movement of lipids and proteins between the apical and basolateral surfaces (review by [28]). Loss of claudin-5 expression results in increased vascular permeability as seen in neurodegenerative diseases such as AD and multiple sclerosis [29]. VE-cadherin is a key endothelial-specific AJ protein that is critical for maintenance of vascular integrity, with reduced VE-cadherin levels or failure of its appropriate positioning in the cell membrane resulting in leaky vessels [30, 31]. VE-cadherin also regulates angiogenesis and EC survival [32, 33].

While brain ECs are the primary cells involved in maintaining the integrity of the BBB, part of this gatekeeping function is also dependent on their interaction with pericytes [34]. This interaction, which is required for BBB maturation, is mediated through platelet-derived growth factor β (PDGFβ) secreted by ECs, binding to the PDGFRβ receptor (PDGFRβ), on pericytes [35]. Furthermore, pericyte-deficient mice show an increase in transcytosis activity in brain ECs [36]. Recently, pericyte dysfunction as seen by abnormal contractility and lack of coverage to support the endothelium has been associated with reduced blood flow and vascular permeability [37].

Aging is defined as a gradual decline in organismal cellular function. One of the major hallmarks of cellular aging is cellular senescence, characterized by permanent cell cycle arrest and specific changes in morphology, metabolism, gene profiles, and activation of a pro-inflammatory secretome, called senescence-associated secretory phenotype (SASP) [38]. Senescence can be induced by triggers such as proliferation, oxidative, and oncogenic stress. Even small numbers of senescent cells can have profound effects on the tissue, through disruption of its architecture and function. Furthermore, SASP factors can impact neighboring cells, inducing senescence through a paracrine action as well as imposing an inflammatory nature on the surrounding tissue [39, 40]. Indeed, senescent cells are a hallmark of chronic inflammation seen in age-associated diseases such as cardiovascular disease, diabetes, and cancer [41]. In vitro replicative stress-induced senescent ECs show increases in the Aβ-40 peptides, although a decreased expression of amyloid precursor protein [42] suggesting a perturbation of the amyloid pathway. Amyloid peptide-induced oxidative stress have also been demonstrated in vitro and post-mortem AD brain tissues [43, 44], which can result in stress-induced premature senescence.

In AD, in both human studies and in animal models, senescent neurons, astrocytes, microglia, and oligodendrocyte precursors have been reported [45–48]. The presence of senescent BBB-related cells has not been described. Here, we investigate the presence of senescent cells in the vasculature of human tissue and in two AD mouse models thereby allowing the temporal investigations into vascular changes. Since both the amyloid precursor protein (APP)/presenilin-1 (PS1) double transgenic and the APP23 single transgenic mouse models are characterized by overproduction of soluble amyloid-β, induction of cellular senescence is likely to be a result of stress-induced premature senescence mediated through this overproduction.

## Materials and methods

### Animals and human brain tissue

All animal experiments were performed according to the Animal Welfare Committee of Royal Prince Alfred Hospital and Animal Ethics Committee of the University of Sydney, Australia. Mice were given food and water ad libitum. Mice were also maintained under specific pathogen-free facility conditions with a 12 light/12 dark cycle. Experimental male and female mice were maintained in separate cages and not more than 6 mice per cage.

Using mouse models that overexpress human amyloid precursor protein gene (APP) with the Swedish mutation and the human presenilin-1 gene with deletion of exon 9 (APPswe/PS1dE9), both familial AD genes, amyloid-β deposition is seen as early as 4–6 months of age, significant amyloid-β deposition by 9 months [49]. This study investigated wildtype and APP/PS1 transgenic littermates at 2 months of age (pre-plaque stage) and 8 months of age (post-plaque formation). APP23 transgenic mice overexpress the same human APP gene carrying the same Swedish mutation. Amyloid-β deposition can be seen at 12 months and size/number of plaques increase is observed at 24 months of age [50]. This study investigated APP23 mice at 3 months (pre-plaque) and 21 months of age (post-plaque formation).

For detection of p16 in vivo, we used the p16-3MR or trimodality reporter mouse that was created by [51]. Briefly, this mouse model contains a single integrated copy of the engineered bacterial artificial chromosome that allows p16INK4a promoter to drive 3MR expression. Its trimodal fusion protein contains reporters such as monomeric red fluorescent protein (mRFP), synthetic Renilla luciferase (LUC), and truncated herpes simplex virus 1 thymidine kinase for deletion experiments. These mice are diploid for p16INK4a and p19-Arf. In this study, we crossed p16-3MR mice with APP/PS1 mice to create WTxp16-3MR and APP/PS1xp16-3MR littermates. Two-month-old littermates were genotyped and those positive for mRFP were used for detection of p16 expression. APP/PS1 mice that were negative for mRFP were used as mRFP negative staining control. All transgenic mice used in this study are on C57BL/6 genetic background.

Fresh frozen blocks of human post-mortem tissue taken from the mid-temporal region from patients with Alzheimer’s disease and non-demented controls were acquired from Sydney Brain Bank with ethics approval obtained by the Human Research Ethics Committee at Royal Prince Alfred Hospital Protocol No. X21-0469 (Table 1).

**Table 1:** Demographics and clinical details of each case

*Age* *(years)*	*Gender*	*Disease duration (years)*	*Clinical diagnosis*
100	M	0	Control
80	F	0	Control
84	F	0	Control
80	F	10	Alzheimer’s disease
85	F	5	Alzheimer’s disease
99	M	3	Alzheimer’s disease

### Cell culture

Human umbilical vein endothelial cells (HUVECs) were isolated as previously described [52] and used from passage 1 to 3. Ethics approval for the collection of the tissue was from SLHD Protocol No. X16-0225. Human cerebral microvascular endothelial cell line (HCMEC/D3) was kindly donated by Prof. George Grau (Bosch Institute, The University of Sydney) and maintained as previously described [53].

### Cellular senescence induction

HUVECs and HCMEC/D3 cells were seeded into 6-well plates or T25 flasks at a density of 240,000 or 350,000 cells, respectively. Next day, the cells were treated with a single dose of 350 μM or 250 μM H_2_O_2_, respectively. Control cells had no treatment of H_2_O_2_. After 24 h, the H_2_O_2_-containing media was replaced with normal HUVEC Dulbecco’s Modified Eagle Medium or Endothelial Basal Medium-2 (Lonza) for the HCMEC/D3 cells. Cellular senescence was monitored over 5 days.

### FITC-dextran transendothelial permeability assay

Control and H_2_O_2_-treated HUVECs or HCMEC/D3 were seeded at 1.0 × 10^5^ or 2.0 × 10^5^ cells, respectively, in 3 μm 24-well polycarbonate Transwell inserts (Corning) pre-coated with 50 μg/mL fibronectin (BD Biosciences) and allowed to attach overnight. One mg/mL FITC-dextran (40 kDa) was added to the upper chamber. The FITC-dextran that diffused through the cell layer into the bottom chamber was collected and measured at 10-min intervals up 60 min and quantified using the POLARstar Omega (BMG LabTech, Mornington, Australia).

### Senescence-associated beta-galactosidase fluorescence staining for cultured cells and tissue sections

Cultured cells were washed once with PBS and trypsinized for 5 min in 37 °C. Cells were pelleted and washed once with PBS. Next, the cells were fixed in 4% paraformaldehyde (PFA) in PBS for 10 min at room temperature. The cells were stained with CellEvent Senescence Green kit (Invitrogen) according to manufacturer’s protocol for 2 h (HUVECs) at 37 °C. Cells were then washed once with 1X PBS supplemented with 10% fetal bovine serum (PBS-10% FBS) and stained using a protocol adapted from [54]. Briefly, the cells were stained overnight with constant mixing with antibodies for senescence such as rabbit anti-human p16 or rabbit-anti human p21 (Table 2). Cells were washed twice with PBS-10% FBS and were then incubated with 1:800 secondary antibodies (Molecular Probes) for 45 min on ice. Next, cells were washed twice and stained with DAPI for 10 min. Finally, cells were washed once more and analyzed by ImageStreamX.

**Table 2:** Summary of antibodies used for immunofluorescence and Imagestream

Antibodies (immunofluorescence)	Source	Dilution
Rat anti-mouse CD31 (MEC13.3)	BD Bioscience	1:200
Rat anti-mouse CD144	BD Bioscience	1:100
Rabbit anti-biotin	Abcam	1:200
Rabbit anti-mouse claudin-5	Invitrogen	1:100
Rabbit anti-mouse PDGFRβ (APB5)	Invitrogen	1:200
Goat anti-mouse albumin	Bethyl Laboratories	1:200
Rabbit anti-human β-Amyloid (D54D2)	Cell Signaling Technology	1:500
Isolectin-B4-biotin	Vectorlabs	1:200
Rat anti-human VE-cadherin	Novus Biologicals	1:100
Rabbit anti-human p16	Origene	1:100
Rabbit anti-human p21	Abcam	1:100
Goat anti-biotin	Invitrogen	1:200
Goat anti-RFP	Rockland	1:200
Rat anti-p21 pre-conjugated Alexa Fluor 594	Santa Cruz	1:200

Two- and 8-month-old APP/PS1 transgenic mice and matched WT controls were anesthetized with ketamine/xylazine and cardiac perfused with ice-cold PBS. After euthanasia, the brains were collected and immersion-fixed with 4% PFA in PBS for 24 h at 4 °C. After fixation, the brains were treated with 30% sucrose in PBS overnight at 4 °C to cryopreserve the tissue. The brains were embedded into optimal cutting temperature (OCT) compound and frozen blocks of tissues were sectioned into 20-μm thick sections using a microtome-cryostat (Leica). Brain sections were brought to room temperature and washed twice with PBS to remove OCT compound. The sections were stained with CellEvent Senescence Green kit (Invitrogen) (senescence green probe (SGP)) according to manufacturer’s protocol for 2.5 h at 37 °C. After incubation, the sections were washed twice in PBS. Finally, these sections were subjected to immunofluorescence staining. As a comparison with SGP staining, brain sections were also stained using traditional senescent β-galactosidase enzyme activity assay kit (Cell Signaling Technology) according to manufacturer’s instructions.

### Immunofluorescence staining in mice

Unless indicated, animals were perfused with cold PBS or 1% paraformaldehyde fixative. After SGP stain, frozen tissue sections were mainly permeabilized and blocked with 1% bovine serum albumin (BSA) in PBS with detergent 0.3% Triton-X100 (PBST-0.3%). Sections were stained overnight with antibodies for blood vessels (Isolectin-B4), endothelial cells (CD31), pericytes (PDGFRβ, CD13), amyloid plaques (D54D2), or albumin leakage (Table 2). After primary antibody incubation, the sections were washed extensively with tris-buffered saline 0.3% Triton-X100 (TBST) and incubated with 1:500 Alexa Fluor 555, Alexa Fluor 594, and/or Alexa Fluor 647-preconjugated secondary antibodies (Molecular Probes, Invitrogen) for 2 h at room temperature. Next, sections were washed three times with TBST and stained with DAPI for 10 min at room temperature. Finally, sections were washed once in PBS and cover-slipped with prolong gold anti-fade mounting medium.

### Immunofluorescence staining in post-mortem human brain tissue

Frozen brain tissues from the mid-temporal cortical region were cut into 8-μm sections. For VE-cadherin staining, the sections were fixed in 4% paraformaldehyde for 10 min. For p21 staining, the sections were fixed in methanol in − 20 °C for 20 min. After fixation, sections were stained as stated above.

### Blood–brain barrier permeability assay using tracer injection

To determine small molecule leak at the pre-plaque stages, we used methods that were adapted from [55]. Briefly, 2- to 3-month-old wildtype and APP/PS1 mice were each injected with 100 mg/kg body weight of unconjugated biotin. The tracer was allowed to circulate in the blood for 1 h before euthanasia. The total period from euthanasia to cardiac perfusion is 10 min per mouse. Mice were perfused through the left ventricle compartment with 10 mL of cold 1X PBS followed by 10 mL 1% PFA/1X PBS. The brain cortices were collected and immersion-fixed with 4% PFA for 5 to 6 h at 4 °C. For detection of vascular leak of biotin and endogenous albumin, 100-μm thick sections were cut using a vibratome (Leica). These tissues were blocked overnight with 1% BSA in PBST-1%. Sections were stained for anti-biotin or albumin, SGP, p21, mRFP, PDGFRβ, and/or VE-cadherin and claudin-5 to determine whether leakage of the tracer was localized to sites of pericyte or endothelial cell senescence, respectively. Vascular leak was determined when leak of either albumin/biotin was detected within the tissue. Albumin/biotin detected at blood vessels located at top and bottom of the Z-stack was excluded as these leaks were considered an artifact of tissue cutting.

### Imaging and image analysis

The stained sections were imaged using a confocal microscope SP8 (Leica). Tilescans of whole brain sections were imaged using × 20 dry objective. Only vasculature in the cortex area was analyzed using ImageJ software (2.30/1.53q). Next, the vessel density or area was analyzed using CD31 expression. The number of SGP vascular cells was counted and expression of SGP determined relative to vascular density. To determine SGP expression and albumin leak in endothelial cells and pericytes, these cells were imaged using × 40 objective and analyzed using the 3D rendering and section tool from ImageJ and Imaris software (version 9.6). The number and size of albumin leaks in the cortical region were measured using ImageJ software and Imaris for each brain section, respectively. The distance of SGP^+^ cells to the nearest area of leak on the blood vessel was measured using 3D rendering and line measuring functions in Imaris software. The correlation of leak size and distance of senescent vascular cells were calculated by assessing each leak for the presence of senescent endothelial cells or other cell types using Graphpad Prism. Since it has been shown that endothelial junctions can differ in expression in selective regions of the brain at during AD progression [56], up to 7 field of view was taken to include different areas of the cortex (front, mid, posterior) and hippocampal region to analyze VE-cadherin and claudin-5 expression in the APP/PS1 and age-matched WT. Fluorescence intensity was measured using WT as baseline level for VE-cadherin and claudin-5 expression. Threshold value was determined using the sample which has the lowest fluorescence signal for either VE-cadherin or claudin-5, i.e., APP/PS1 samples. Expression of claudin-5 and VE-cadherin were measured as mean fluorescence intensity relative to area of vasculature using ImageJ software.

Negative staining controls for immunofluorescence staining are included in Fig. s11.

### Imaging flow cytometry analysis

To validate the SGP as a marker for senescence in primary human, the ImageStream X.2 (Amnis) system was used that combines features of fluorescent microscopy and quantitative features of flow cytometry. Images of H_2_O_2_-induced senescent HUVECs and untreated controls were acquired at × 20 magnification. At least 2000 cells were used for each color to generate the compensation matrix. At least 2000 unstained cells were also collected as negative control. A total of 50,000 cells were collected per experimental group and analyzed using the IDEAS software version 6.2 developed by Amnis. Cells were gated for focused cells using the contrast and gradient RMS features of brightfield (BF) images. Next, cells were gated for single cells using aspect ratio and area of BF images. After that, cells that were DAPI positive were selected for gating. Senescent and non-senescent cells were gated for using the size or area of BF image, mean intensity of SGP, and either p16 or p21.

### Isolation of mouse brain vasculature

Blood vessels were isolated using a modified form of the method of [10]. Two-month-old APP/PS1 transgenic mice and WT littermates were euthanized and perfused with cold PBS. Brains were collected and the meninges removed in cold PBS containing 2% FBS. The cortex and hippocampus were homogenized with a glass pipette in 2% FBS. The homogenized brain was mixed with dextran (Sigma) to a final concentration of 16% and centrifuged at 4000* g* for 30 min. The dextran gradient produced a pellet enriched with blood vessels at the bottom and a top layer of myelin and brain parenchymal cells. The top layer was removed and the pellet was washed over a 40-μm strainer with cold PBS. The blood vessels remaining on the strainer were collected in PBS and pelleted for RNA extraction.

### RNA extraction, cDNA synthesis, and quantitative PCR

Total RNA was extracted using Trizol reagent (Ambion, Life Technologies) from mouse brain microvessels. A total of 1 µg/µL of total RNA was reverse transcribed using the high-capacity cDNA reverse transcription kit (Applied Biosystems) and cycled in the BIO-RAD T100™ Thermal Cycler. Real-time PCR were performed for the analysis of gene expression using PowerUp™ SYBR™ Green Master Mix (Applied Biosystems) with 10 μmol of forward and reverse primers. The list of primers for each gene can be found in Table 3. Quantification of the gene of interest was calculated relative to the tubulin control gene by the comparative (∆∆C_T_) method.

**Table 3:** List of mouse primers

Gene	Forward primer	Reverse primer
*Tubulin (Tuba1a)*	ctggaacccacggtcatc	gtggccacgagcatagttatt
*VE-Cadherin (Cdh5)*	gttgccacatctcagggaat	ccttcctccagctgtcactc
*Claudin****-****5 (Cldn5)*	cgggtgagcattcagtcttt	gtcacgatgttgtggtccag
*Tie2 (Tek)*	ccctcctcaaccagaaaaca	tctaggcccttgagctggta
*p21 (Cdkn1a)*	ttgccagcagaataaaaggtg	tttgctcctgtgcggaac

### Statistical analysis

All statistical analysis was performed using Graphpad Prism version 9. In experiments in which a single experimental group was compared with a single control group, statistical comparisons were made by an unpaired *t*-test. All comparisons were 2-tailed unless otherwise indicated. Pearson’s correlation coefficient was used for all correlation analysis. A *p*-value of less than 0.05 was considered significant. Data displayed in figures with error bars represent the mean ± SD.

### Isolation of primary mouse endothelial cells for single cell RNA sequencing

Brain dissociation and endothelial cell isolation was performed using the adult brain dissociation kit and CD31 magnetic beads from Miltenyi Biotec. Briefly, meninges were removed mechanically and brain cortex from 9-month-old APP/PS1 transgenic and WT mice were collected into ice-cold PBS. After magnetic bead separation, the flow through containing endothelial cells was subjected to single-cell RNA sequencing (scRNA seq) using 10X chromium.

### Single-cell RNA sequencing quantification and statistical analysis

#### Read alignment and expression count table generation

From the sequencing results of the 10 × chromium experiments, the unique molecular identifiers, cell barcodes, and the genomic reads were extracted using cell ranger with default parameters (v3.1, 10 × Genomics). The extracted reads were aligned against the annotated mouse genome, including the protein and non-coding transcripts (GRCh38, GENCODE v27). The reads with the same cell barcode and unique molecular identifier were collapsed to a unique transcript, generating the count matrix where columns correspond to single cells and rows correspond to transcripts. To remove potentially unhealthy or suboptimal cells, cell filtering was performed using the number of reads, the proportion of genes expressed, and the fraction of mitochondrial reads as criteria. Specifically, cells with greater than 99% of genes not expressed and 10% of mitochondrial gene expression were removed. Transcripts from mitochondrial- and ribosomal-protein coding genes were discarded for downstream analyses such as embedding and clustering, because they are typically known to be highly expressed irrespective of biological identity.

#### Doublet detection and filtering

The presence of multiplets in single-cell data can arise from incomplete dissociation of single cells meaning that more than one cell can be encapsulated in GEMs. DoubletFinder, an algorithm to detect multiplets in single-cell data, was used to remove potential doublets or multiplets from each biological batch at a threshold of 10% [57].

#### Dimensionality reduction, clustering, and cell-type annotation

To embed the single-cell transcriptomes into the latent space, we first performed principal component (PC) analysis on the binomial Pearson’s residuals calculated using the scry package [58] on the feature-selected count matrix. Feature selection was performed using scry, and the top 1500 genes were used. Using the first 40 PCs, the reduced embeddings were generated by using fftRtsne, the FFT-accelerated interpolation-based tSNE from the FIt-SNE package [59]. The first 35 PCs were used to construct the shared nearest neighbor graph using the default arguments of the buildSNNGraph function in the scran R package [60] with which the graph-based Louvain clustering was performed to generate the final clusters. Cell identity analysis was performed using the Cepo R package [61]. Finally, annotation of the clusters from the in-house datasets was performed by manual assignment of cell types through inspection of cell identity genes.

#### Differential gene expression analysis

Differential expression analysis between healthy and diseased cells was performed using limma [62], conditioning on cell type. Significance was determined as the FDR-adjusted *p*-value lower than 0.05 and a log2 fold-change lower than − 0.1. The results were visualized as a volcano plot using the EnhancedVolcano R package; the overlap among gene sets were visualized as a Venn diagram using the ggVennDiagram R package [63].

#### Gene set over-representation analysis of maturation-associated genes

Gene set over-representation analysis was performed on the genes significantly downregulated in healthy and diseased mouse brain. The gene set enrichment analysis was performed for each cell type for healthy and disease gene sets. Using the GO terms related to biological processes relating to “ADHESION” and the “BLOOD BRAIN BARRIER” from the C5 ontology gene set from the MSigDB collection [64], we assessed the over-representation of these gene sets among the differentially downregulated genes using the fgsea R package [65].

#### Derivation of signature of adhesion and blood–brain barrier activity

To generate a signature score denoting the activity of adhesion and BBB associated pathways, we performed differential gene expression analysis using the limma pipeline [62]. To identify the set of genes that can be used to quantify the dysregulation in the blood brain barrier activity during AD, we selected the genes that are significantly downregulated in AD compared to wild-type control. Then, among these significantly regulated genes, we intersected the genes associated with the blood brain barrier and adhesion. After filtering for these genes, we arrived at the final 80 genes (Tab. s1) which we used to determine the signature score for BBB activity for each cell.

#### Visualization of gene expression

Normalized gene expression was visualized as a dot plot or split violin and box plots. Within the box plots, the median is denoted as red circles. The box denotes the inter-quartile range. Outliers have been omitted to facilitate visualization. The student’s *t*-test was used to compare the difference in mean gene expression between healthy and diseased cells. ns, *p*-value > 0.05; *, *p*-value ≤ 0.05; **, *p*-value ≤ 0.01; ***, *p*-value ≤ 0.001; ****, *p*-value ≤ 0.0001. The ggplot2 and introdataviz R packages were used for visualization [66, 67].

## Results

### Characterization of a fluorescence-based senescence associated beta-galactosidase, as a senescent marker for *in vitro* and *in vivo* use

Senescence is commonly identified by a range of markers, including an increase in lysosomal senescence-associated β-galactosidase activity (SA-β-gal), upregulation of the cell cycle markers, p16 and p21, and an increase in cell size. The traditional method for detection of SA-β-gal uses enzyme-catalyzed hydrolysis of β-galactosidase. However, the blue compound is not fluorescent, thus making it unsuitable for use in conjunction with fluorescent cell-type-specific markers. Therefore, we validated a newly developed fluorescence-based senescence-associated β-galactosidase assay (SGP).

**Fig. 1:**
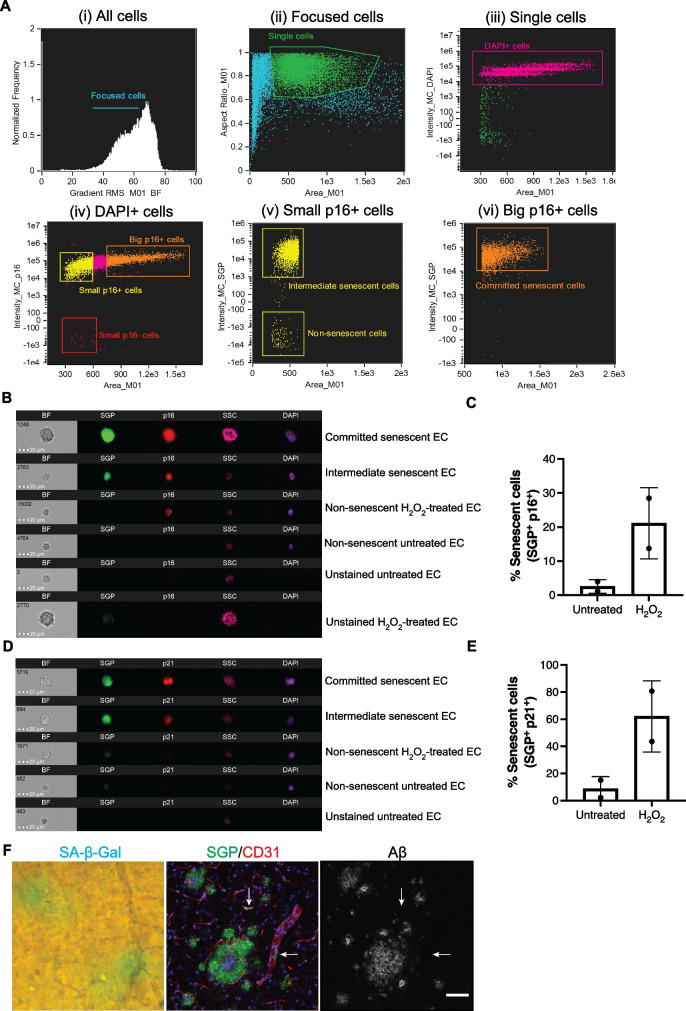
Validation of senescence green probe (SGP) as a marker of senescence. **Ai-vi** shows a representative gating strategy used to identify senescent cells populations in H_2_O_2_-treated cells. Focused cells were gated into single cell population (green box). DAPI^+^ cells (magenta box) was analyzed for p16 expression followed by SGP expression against cell area/size. Small p16^+^ cells (yellow box) shows a population with high SGP expression (intermediate senescent cells) and another population negative for SGP expression (non-senescent cells). A majority of the large cells with high expression of p16 (orange box) also had high expression of SGP (committed senescent cells). Untreated, H_2_O_2_-treated, and unstained HUVECs were analyzed by Amnis ImageStream and gated in the same fashion. **B** Representative images of unstained HUVECs, non-senescent HUVECs, and HUVECs at different stages of senescence according to their cell size in brightfield (BF) and side scatter (SSC) as well as expression of senescent markers SGP (green) and p16 (red). Committed senescent cell in the representative image shown here has two DAPI (purple) positive nucleus. **C** Quantification of the percentage of SGP^+^ and p16^+^ large senescent cells in untreated and H_2_O_2_-treated HUVECs. **D** Representative images of HUVECs at different stages of senescence according to cell size and senescent markers SGP (green) and p21 (red). **E** Quantification of the percentage of SGP^+^ and p21^+^ large senescent cells in untreated and H_2_O_2_-treated HUVECs. In vitro data presented for p16- or p21-independent experiments are from 2 different HUVEC lines and are presented as mean ± standard deviation, scale bar = 20 µm. **F** Representative images of traditional SA-β-gal (blue) and SGP (green) stain in the brain taken from APP/PS1 mice after amyloid plaque development. Amyloid beta (gray) staining shows that SGP (green) staining on CD31^+^ vasculature (red) is localized to vascular cells, not plaques deposited on the blood vessel. Scale bar = 50 µm

Senescence was induced in human umbilical vein endothelial cells (HUVECs) and human cerebromicrovascular EC line (HCMEC/D3) using sub-lethal doses of hydrogen peroxide (H_2_O_2_), a routinely used in vitro model for oxidative stress-induced senescence [68] also noting that oxidative stress has been linked to AD [43, 44, 69]. After 5 to 8 days of a single dose of H_2_O_2_, the ECs take on an enlarged and flattened morphology (Fig. s1A). These large cells also had high nuclear expression of the cell cycle kinase inhibitor protein p21 or SGP expression confirming their senescent phenotype (Fig. s1A&B). Quantification of the senescence based on their cell size and expression of SGP showed untreated HUVECs and HCMEC/D3 cell line have 3.6% and 4.4% senescence, respectively. H_2_O_2_-treated HUVECs and HCMEC/D3 cells have 37% and 29.5% senescence, respectively. These results show that H_2_O_2_ induction of premature senescence is comparable between HUVECs and the brain ECs, HCMEC/D3 cell line. Quantification of SGP positivity was analyzed in HUVECs using Amnis Imagestream, as detailed in the “Materials and methods” section. ECs were fixed and stained as a cell suspension with 4’, 6-diamidino-2-phenylindole (DAPI), SGP, and p16. Untreated and H_2_O_2_-treated ECs with no staining were used as negative controls. Figure 1A shows the gating strategy that was used for identifying senescent ECs. Blurry images of single cells were excluded, and only focused cells were collected for analysis and further gated for single cells. Finally, single cells positive for DAPI were differentiated from cellular debris. Senescence was determined in the DAPI-positive population using the mean intensity of p16 and cell size. Cells that were gated as big cells and positive for p16 were found to be high in SGP expression. These cells are likely to be committed senescent cells (Fig. 1A & B), whereas cells that were small but high in p16 and SGP expression are likely to be intermediate (or pre-committed) senescent ECs (Fig. 1A and B). Figure 1B also shows the morphology of non-senescent ECs, which were small and had no detectable SGP and p16 expression. Unstained ECs showed no staining for SGP, p16, and DAPI. Figure 1C shows that the percentage of senescent ECs, as defined by enlarged cell size and high p16 and SGP expression, was increased in H_2_O_2_-treated ECs compared to untreated ECs, to a similar magnitude as previously reported using conventional immunohistochemistry staining for SA-β-gal [52]. In addition, SGP was also found to be highly co-expressed with high expression of nuclear associated p21 in large senescent ECs (Fig. 1D). There was a seven- to eightfold increase of SGP/p21 in senescent H_2_O_2_-treated ECs compared to untreated controls (Fig. 1E). The percentage of SGP/p21 was higher compared to SGP/p16 in senescent H_2_O_2_-treated ECs likely reflecting the stage of senescence as has previously been reported in replicative senescent human diploid fibroblasts [70]. In the early stage, the cells have just entered cell-cycle arrest and were suggested to be pre-senescent or at an early stage of senescence. At late stage or deep senescence, p16 expression increased and there was a decrease in p21 levels. These changes have been associated with further enlargement of cell size and SA-β gal activity [71]. Our in vitro model is only an acute experiment of oxidative stress-induced senescence taken at 5 to 6 days and shows that the majority of the senescent cells express high levels of p21 and with a lower percentage expressing p16, indicative of most cells still being in the early pre-senescent stage. The SGP expression was further tested in vivo in fixed APP/PS1 mouse sagittal brain sections and compared with conventional SA-β gal stain. Both SA-β gal and SGP had similar expression patterns in whole brain sections, where it was seen associated with amyloid plaques (Fig. s2A&B) in 6- to 8-month-old APP/PS1 mice similar to previously reported by Zhang and colleagues [48]. Staining with a pan-amyloid-β marker showed that SGP also associated with amyloid plaques (Fig. 1F).

Our results show that SGP staining identifies senescent cells both in vitro and in vivo. Co-staining with CD31 as a marker for blood vessels revealed that SGP expression is also localized to the vasculature (Fig. 1F, white arrows) and can be distinguished from amyloid plaques in APP/PS1 brain sections. Unfortunately, commercially available antibodies against p16 and p21 on mouse tissues have been extremely unreliable, as previously reported [51, 72].

### Vascular senescence is present in mouse models of amyloidogenesis

**Fig. 2:**
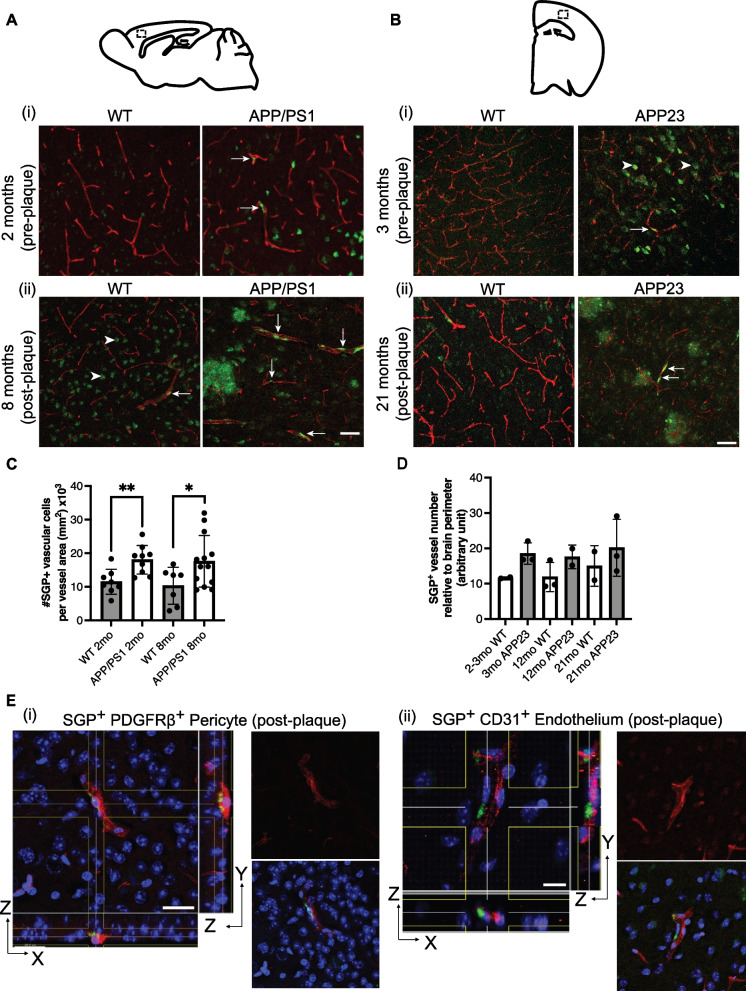
SGP expression in mouse models of AD. Representative images of SGP expression in APP/PS1 (**A**) and APP23 (**B**) compared to wildtype (WT) mice over different stages of plaque development. **Ai** and **Bi** White arrows show the presence of vascular cells positive for SGP (green) in 2-month-old APP/PS1 and 3-month-old APP23 mice that have no detectable plaques. **Aii** and **Bii** Representative images show that more SGP-positive vascular cells (white arrows) can be detected in APP/PS1 and APP23 mice after plaque formation compared to controls. Arrowheads in **Aii** and **Bi** show non-vascular cells that have SGP expression. **C** and **D** Quantification of the number of SGP-positive cells on the vasculature relative to brain cortical area in APP/PS1 and APP23 mice, respectively. Data in **C** is from 3 independent experiments and represented as mean ± standard deviation. The Mann–Whitney *t*-test was used to determine difference in SGP expression between WT and APP/PS1 transgenic mice. *p-value ≤ 0.05, **p-value ≤ 0.01, WT = 5 per age group APP/PS1 = 8–10 per age group. Data in **D** is represented as mean ± standard deviation, WT = 2–3 per age group and APP23 = 2–3 per age group. **Ei** Localization of SGP (green) in pericytes stained with PDGFRβ (red) in large and small vessel. Scale bar = 20 µm. **Eii** Localization of SGP (green) found in the endothelium stained with CD31 (red). Scale bar = 15 µm

We used APP/PS1 mice to investigate the development of vascular senescence with age. Pan-amyloid-β staining showed the absence of amyloid in 2-month-old APP/PS1 mice and significant amyloid formation in 8-month-old APP/PS1 mice (Fig. s3). In 2-month-old WT mice, there were only small numbers of SGP^+^ vascular cells detected and minimal SGP^+^ non-vascular cells (Fig. 2Ai). However, SGP expression was increased particularly in non-vascular cells in 8-month-old WT (arrow-heads, Fig. 2Aii). In the APP/PS1 mice, at 2 months of age, there were increased in senescent cells associated with the vasculature (Fig. 2Ai, white arrows). SGP expression on the vasculature was measured as the number of SGP^+^ vascular cells relative to vascular density or vessel area. Our analysis showed that there was a significant increase in the SGP^+^ cells first in 2-month-old APP/PS1. This increase was maintained at 8 months compared to their WT controls (Fig. 2C). A similar trend was observed in a second mouse model with amyloid deposition, the APP23 strain, at 3, 12, and 21 months, where SGP^+^ vascular cells were increased compared to age-matched WT mice (Fig. 2B&D). We also observed non-vascular cells positive for SGP before plaque development in the 3-month-old APP23 mice (Fig. 2Bi, arrowheads). SGP was also localized to amyloid plaques (as can be seen in Fig. 1F), likely because senescent oligodendrocytes are known to be present in the amyloid plaques, as was demonstrated previously [48].

To identify cell types on the vasculature that were SGP^+^, we used the pericyte marker PDGFRβ and the EC marker CD31 and found that both pericytes and EC were identified as SGP^+^ in 8-month-old APP/PS1 mice (Fig. 2E).

### Localized vascular leak detected after plaque formation is associated with vascular senescence

Cultured senescent HUVECs and HCMEC/D3 cells show a decrease in the endothelial AJ molecule, VE-cadherin, and tight junctions claudin-5 and ZO-1 (Fig. s1B). Consistent with this, they have an increased permeability under basal conditions (Fig. s1C&D). We therefore sought to investigate whether the senescent cells seen in the vasculature of AD mice were associated with changes in BBB integrity as determined by vascular leak. We used post-mortem detection of endogenous albumin to determine transcellular-mediated leaks [73].

**Fig. 3:**
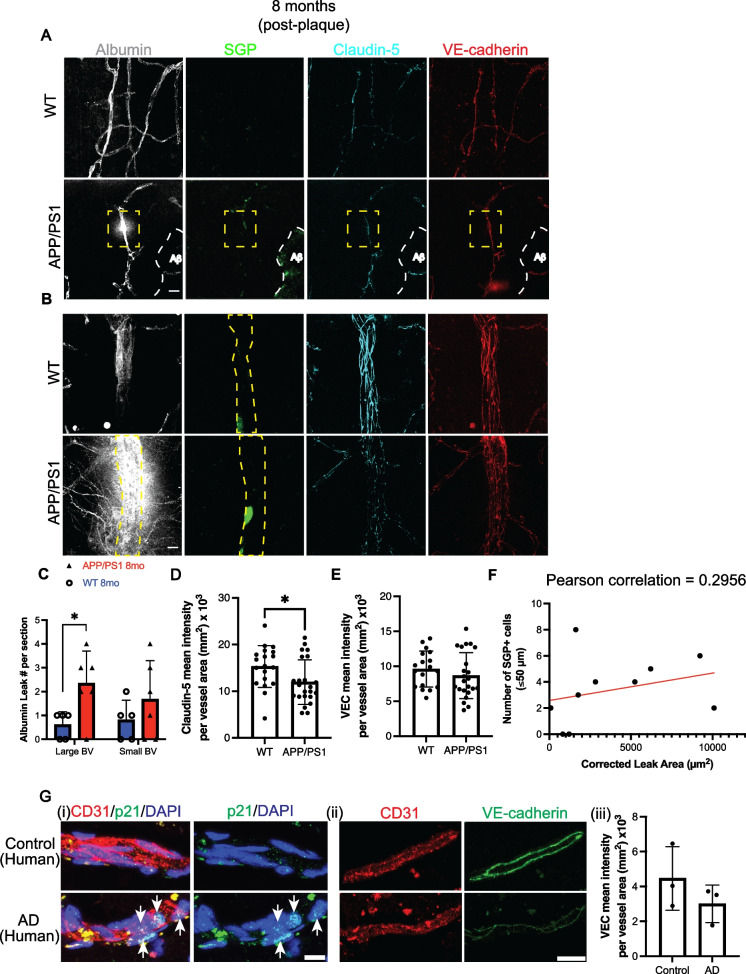
Association of albumin leak and endothelial junctions with SGP + vascular cells after plaque formation. **A** Representative image of SGP^+^ EC (green) localized to a capillary leak of endogenous albumin is indicated by dashed yellow box. Amyloid plaques were observed in 8-month-old APP/PS1 mice (white dashed lines). Claudin-5 (cyan) and VE-cadherin (red) expression appear to be reduced and disorganized, respectively. **B** Representative image of SGP^+^ perivascular cell (green) localized to large vessel leak of endogenous albumin. No leak was observed in similar vessel in WT mice. **C** Albumin leak was found more in large and small blood vessels (BV) in older APP/PS1 mice (red) compared to wildtype (WT) mice (blue). **D** Global Claudin-5 expression was significantly reduced in APP/PS1 mice compared to WT mice. **E** VE-cadherin was not significantly altered between 8-month-old WT and APP/PS1 mice. **F** Positive correlation of number of SGP^+^ cells within 50 microns to leak area in 8-month-old APP/PS1 mice. **Gi** Staining of blood vessels positive for CD31 (red) and senescence marker p21 (green) and nuclei (DAPI, blue) in post-mortem human mid-temporal cortical sections. Senescence of endothelial cells and perivascular cells were detected in human Alzheimer’s disease (AD) brain sections (white arrows). **Gii** Human brain sections had decreased expression of VE-cadherin (green) in blood vessels positive for CD31 (red). **Giii** Overall expression of VE-cadherin in human AD mid-temporal region. Mouse data is from 3 independent experiments; human data is from 3 non-demented controls and 3 AD age and sex-matched cases. All data are represented as mean ± standard deviation. Unpaired *t*-test was used to determine leak number per section, VE-cadherin, and claudin-5 expression between WT and APP/PS1 mice. *p-value ≤ 0.05, WT = 4–6, and APP/PS1 = 5–7 mice. Scale bar in Fig. A = 10 µm, Fig. Gi = 10 µm and Gii = 20 µm

Whole brain sections from WT mice showed little or no leaks of albumin (Fig. s4A). However, there was significant albumin leak, a measure of transcellular leak, in the APP/PS1 mice at 8 months of age (Fig. 3A–C), where quantification of vascular leaks was assessed only in whole blood vessels excluding cut or incomplete vessels from the analysis. Some of these areas of leak were at sites of SGP^+^ ECs in a small cortical vessel (Fig. 3A, yellow dashed box). Larger areas of albumin leak were found at larger blood vessels (Fig. 3B) and adjacent to cells with strong expression of SGP as confirmed by Z-stack analysis (not shown). Regardless of the type of senescent cell associated with the leak or the size of the vessel, they had severe disruption in claudin-5 and VE-cadherin at the site of the leak (Fig. 3A&B). In addition, analysis of the mean fluorescence intensity over the entire vessel showed a significant reduction in the expression of claudin-5 (Fig. 3D). However, VE-cadherin expression in the older APP/PS1 mice showed no significant change compared to age-matched WT mice (Fig. 3E).

Correlation analysis showed a trend towards a positive correlation between vascular leak size and number of SGP^+^ vascular cells within 50 microns to the leak area (Fig. 3F), although it did not reach statistical significance.

For investigations into human post-mortem brain tissue, we used p21 for detection of senescent cells, as it is a reliable marker in human ECs (Fig. 1D & Fig. s1). Analysis of the mid-temporal cortical region showed the presence of senescent p21^+^ EC and perivascular cells (Fig. 3Gi). In the same region, there was often a significant decrease in VE-cadherin (Fig. 3Gii) and a trend towards an overall reduction in VE-cadherin expression across the entire AD tissue (Fig. 3Giii).

### Single-cell RNA sequencing data of isolated ECs from APP/PS1 brains shows expression of cell–cell adhesion pathways that is significantly compromised

**Fig. 4:**
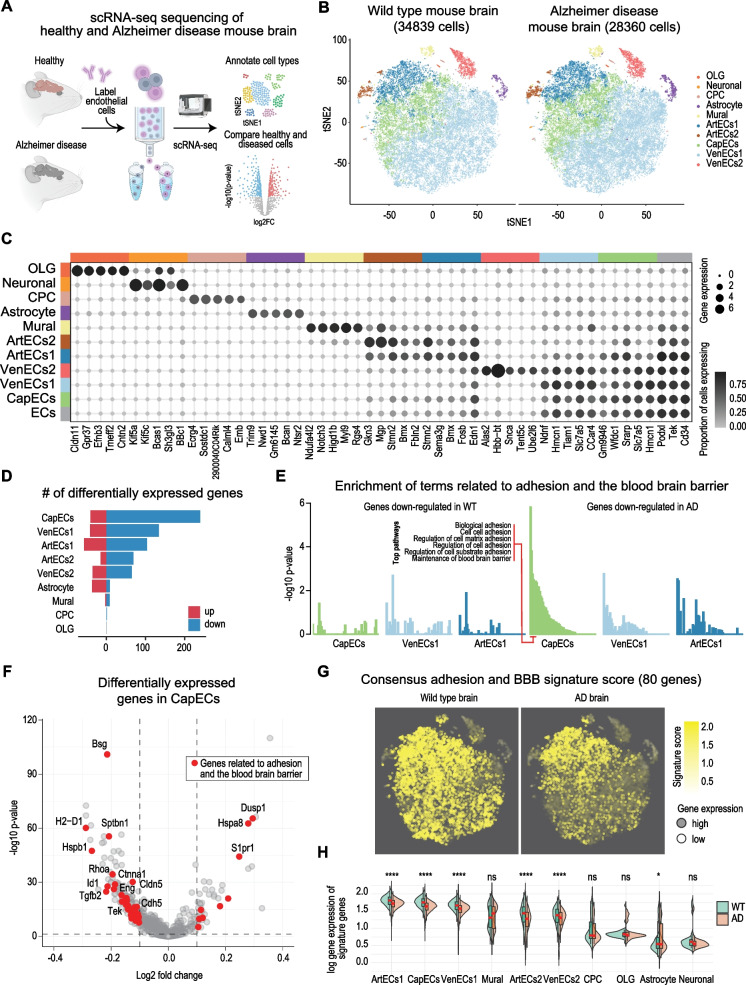
Pathways associated with adhesion and the blood-brain barrier are dysregulated in endothelial cells of Alzheimer’s disease mice. **A** Schematic of single-cell transcriptomic profiling of mouse brain (generated using BioRender). **B** tSNE representation of the transcriptomes of single cells. Cells are colored by their type in healthy (left) and diseased brain (right). OLG, oligodendrocytes; CPC, choroid plexus cells; ArtEC, CapECs, and VenECs, arterial, capillary, and venous endothelial cells. **C** Expression pattern of cell type marker genes. The size of circles denotes the average gene expression across cells and the color denotes the proportion of cells expression the gene. **D** Bar plot showing the number of significantly upregulated (red) and downregulated (down) genes in Alzheimer diseased versus healthy brain. **E** Bar plot of gene set over-representation of pathways associated with adhesion and the blood brain barrier among genes significantly downregulated in healthy (left) and diseased (right) mouse brain for each cell type. *Y*-axis denotes the degree of enrichment, and the *X*-axis denotes the pathways. **F** Volcano plot showing genes differentially regulated in capillary endothelial cells (CapECs). *X*-axis denotes the log2 fold-change in gene expression, and the *Y*-axis denotes the − log10 *p*-value (FDR-adjusted). **G** Visualization of the signature score denoting the transcriptional activity of 80 adhesion and blood-brain barrier (BBB)-associated genes on tSNE. Cells are colored by the signature where a stronger yellow color denotes higher activity, and an opaquer color denotes higher average gene expression. **H** The average normalized gene expression of the signature genes in healthy (WT, green) and Alzheimer’s disease (AD, red) brain cells is visualized as split violin and box plots for each cell type. The Student’s *t*-test was used to compare the difference in mean gene expression between healthy and diseased cells. ns, *p*-value > 0.05, *p-value ≤ 0.05; **p-value ≤ 0.01; ***p-value ≤ 0.001; ****p-value ≤ 0.0001

In order to investigate the global transcriptomic changes, we performed single-cell RNA sequencing (scRNA-seq) of ECs from 8-month-old WT and APP/PS1 mice (Fig. 4A). We profiled 63,199 single cells from brain samples enriched for ECs from female and male mice (Fig. s5). We then performed clustering and manual cell-type annotation into 10 cell types using known markers (Fig. 4B&C). As expected of the EC-enriched samples, the major cell type in the scRNA-seq dataset were ECs (expressing the canonical endothelial markers Tek, Podxl, and CD34), which we further subcategorised into ECs originating from the arterial (Bmx), venous or the capillary (Wfdc1, Hmcn, Slc7a5) vasculature [74–76].

Since our staining had identified changes in VE-cadherin and claudin-5 in the APP/PS1 mice, we investigated whether the adhesion molecule pathways were more downregulated in ECs. We first performed a differential gene expression analysis between the control and diseased samples using the Limma package [62]. We observed that the ECs were among the most regulated cell types, with capillary ECs (CapECs) demonstrating the highest number of regulated genes (Fig. 4D). Interestingly, we found that in general, more genes were downregulated than upregulated, suggesting that transcription tends to be repressed rather than activated in APP/PS1 mice (Fig. 4D). We also noted that the downregulated transcriptional profiles demonstrated a high overlap of 11–28% between the major EC cell types (Fig. s6).

Next, using these gene sets, we performed gene set over-representation of terms associated with adhesion and the BBB. We found that consistent with our findings shown in Fig. 3, the gene sets downregulated with vascular senescence in ECs were significantly enriched for many adhesion and BBB terms (Fig. 4E). While the enrichment was strongest for CapECs, terms such as “Biological adhesion” and “Cell–cell adhesion” were significantly enriched across all the major EC cell types. Moreover, visualization of differentially expressed genes in CapECs confirmed the reduction of key genes, VE-cadherin, and claudin-5 (Fig. 4F), which reciprocated our analyses from the fluorescence imaging (Fig. 3A). To obtain a signature score denoting the overall activity of adhesion and BBB genes, we next calculated the average transcriptomic profile of all regulated genes related to these pathways. This scoring approach enabled us to assign each cell with a score of adhesion activity. Visualization of these scores on UMAP highlighted the extent of the reduction of adhesion/BBB activity in the APP/PS1 mice compared to WT (Fig. 4G). We further demonstrated that this decrease in adhesion/BBB activity is largely specific to ECs in terms of all the signature genes (Fig. 4H) and exemplar genes (Fig. s7). Overall, our single-cell analyses show that the BBB is compromised in its integrity as judged by a decrease in the expression of the EC adhesion molecules notably, VE-cadherin, and claudin-5.

### Vascular leaks are detected before plaque formation.

**Fig. 5:**
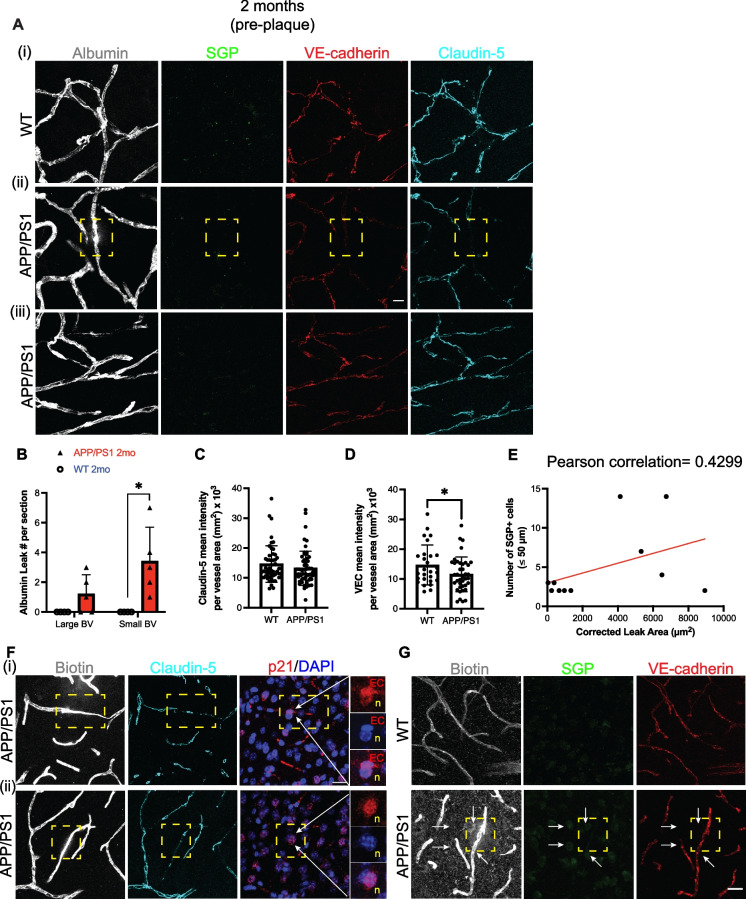
Association of vascular leak and endothelial junctions with SGP + vascular cells before plaque formation. **Ai** Representative image of normal capillary with endogenous albumin restricted to the microvessel in the 2-month-old WT mice. **Aii** Representative image of capillary leak of endogenous albumin (white) is indicated by the dashed yellow box is observed in 2-month-old APP/PS1 mice not associated with SGP^+^ cells. A lack of VE-cadherin (red) and claudin-5 (cyan) expression is observed at site of leak. **Aiii**,** F** In areas of no leak, VE-cadherin is also evidently decreased. **B** Albumin leak was localized to small blood vessels compared to large blood vessels (BV) in APP/PS1 mice (red) compared to wildtype (WT) mice (blue). **C** Image analysis of claudin-5 shows that there is no difference in expression between APP/PS1 and WT littermates. **D** Global VE-cadherin expression is decreased in APP/PS1 mice compared to WT mice. **E** Correlation analysis of leak size and number of SGP^+^ cells within 50 microns to the area of leak in APP/PS1 mice. **F** Capillary leak of biotin (white, dashed yellow box) is also observed, where there is a reduced expression of claudin-5. P21 expression (red) was identified in the DAPI + nuclei (blue) of endothelial cell (EC) and neurons (n) at areas of biotin leak. **G** Capillary leak of biotin (white, dashed yellow box) is also observed, where there is lack of VE-cadherin (red) expression. Data is from 3 independent experiments and represented as mean ± standard deviation. Unpaired *t*-test was used to determine leak number per section, VE-cadherin, and claudin-5 expression between WT and APP/PS1 mice. *p-value ≤ 0.05, WT = 4–6, and APP/PS1 = 5–7 mice. Scale bar in Fig. A = 10 µm, and scale bar in Fig. F and G = 20 µm

To investigate the possibility that vascular leak and senescence are early events in AD pathology, we interrogated large and small vessels in the cortex, before visible plaque formation (2 months of age). Leakage of albumin was predominantly found in the cortical microvasculature in the 2-month-old APP/PS1 mice (Fig. 5A and B). This is in contrast to the 8-month-old APP/PS1 mice where albumin leak was seen in large vessels (Fig. 3C). The size of the leak was also larger in the 8-month mice. The average size leak for 2 months was 1908.8 μm^2^ (*n* = 4) and for 8 months was 3265 μm^2^ (*n* = 9). At the site of albumin leak, there was a decrease in the expression of claudin-5 and VE-cadherin (Fig. 5A, C, and D). However, when the analysis was broadened to whole brain regions, there was a significant decrease in the overall level of VE-cadherin but not of levels of claudin-5 expression (Fig. 5C and D). Correlation analysis showed that the size of the leak is influenced by the number of cells in close proximity (Fig. 5E). Thus, the bigger the leak, the more SGP^+^ cells are within 50 microns away.

In the pre-plaque stage, small biotin leaks into the brain parenchyma were also detected in microvessels (Fig. 5F and G) as well as in large vessels (Fig. s4B). Furthermore, high expression of p21 was observed in an EC (Fig. 5Fi) and neurons (Fig. 5Fii) at two different sites of biotin leak. These cells were identified according to their nuclear shape and location in or around the leaky microvessel. EC nuclei was oval and elongated whereas neuronal nuclei were round, large, and had multiple nucleoli. While SGP^+^ cells were not seen specifically at the leak site (Fig. 5A and G, yellow box), SGP^+^ cells (white arrows, Fig. 5G) were observed in close proximity. Similar to albumin leak, there was a decrease in the expression of claudin-5 and VE-cadherin at the site of biotin leak (Fig. 5F and G).

Our results demonstrate that both the paracellular (biotin) and the transcellular (albumin) pathways are disrupted at the pre-plaque stage. However, we could not assess whether both albumin and biotin are leaking from the same site in the microvessel.

### Pericyte changes in disease development.

**Fig. 6:**
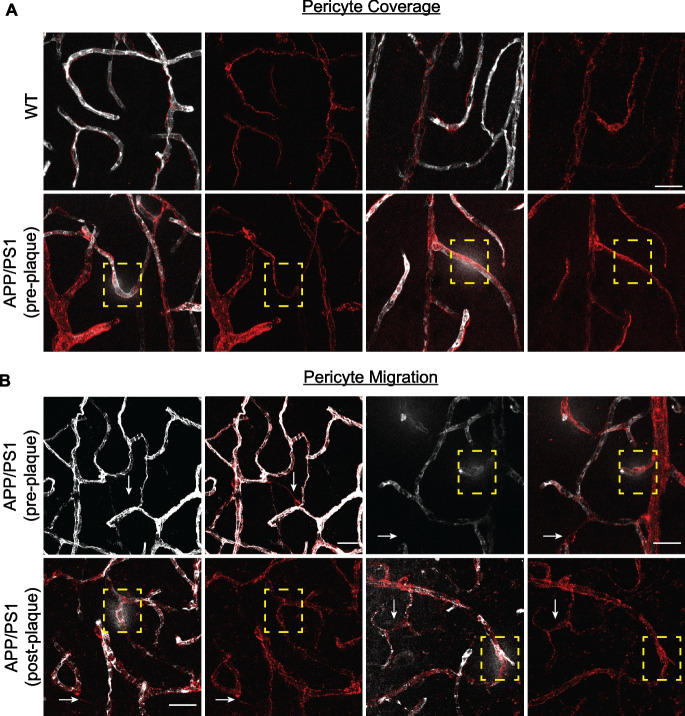
Pericyte morphology at sites of albumin leakage. **A** Representative images of PDGFRβ-positive pericytes (red) showing pericyte coverage in comparison to wildtype (WT) mice, even at sites of albumin leak (yellow dashed box) in the brain parenchyma of APP/PS1 mice before plaque development. **Bi** Pericytes can be detected migrating between the microvessel in the APP/PS1 mice (white arrows) before and after plaque formation. Migrating pericytes were characterized by the extension of pericyte cell body and processes towards another neighboring microvessel, which is outlined by vasculature containing albumin (white). **Bii** After plaque formation, pericyte coverage and migration were still detected on multiple sites of albumin leak (yellow dashed box). Scale bar = 20 µm

The integrity of the BBB is also dependent on pericytes. Examination of the PDGFRβ^+^ pericyte coverage at the sites of vascular leak in 2-month-old APP/PS1 brains (yellow dashed box) appeared normal (Fig. 6A). However, pericyte migration, as visualized by the lifting or extending of the pericyte cell body and its single process towards another proximal microvessel, was observed nearby the vascular leak areas (yellow dashed box) in APP/PS1 mice (Fig. 6Bi, white arrows). The migration of PDGFRβ^+^ pericytes was also observed at vascular leak areas in APP/PS1 mice after plaque formation (Fig. 6Bii).

Next, as SGP expression was not observed in the pericytes before plaque formation in the APP/PS1 mice (data not shown), we used other markers such as p21 and p16. Immunofluorescence staining showed that p21 expression was found mainly in neurons (yellow box, Fig. s8A). Furthermore, p21 expression can also be identified in endothelial cells (red box) and pericytes (green box) (Fig. s8A) via co-staining with PDGFRβ and biotin. Perivascular cells were also found to express p21 (white arrows, Fig. s8A). The expression of p21 was variable among the different cell types. For example, certain neurons expressed higher p21 than other neurons. Biotin leak, however, was not observed in these areas of p21 expression. Interestingly, our results show an increase in p21 expression in the 2-month-old APP/PS1 mice compared to age-matched WT (Fig. s8B).

Littermates from the crossbreeding of APP/PS1 mice with the p16-3MR mice were used to determine p16 expression using the mRFP reporter. Analysis for p16 expression showed that some but not all non-migrating pericytes on microvasculature can express p16 at 2 months (Fig. s9A&b). Co-staining with CD31 also identified certain capillaries to be high in p16 expression. These ECs were also positive for SGP expression (Fig. s9a). In contrast, p16^+^ neurons did not colocalize with SGP expression (Fig. s9c). Negative staining was observed in APP/PS1 mice that was negative for mRFP indicating p16 expression to be specific in the p16-3MRxAPP/PS1 and p16-3MRxWT (Fig. s9d).

Interestingly, there was no colocalization of p21 or p16 in the migrating pericytes (data not shown). These observations show that vessel-associated pericytes may be in a pre-senescent or senescence state before amyloid plaque formation. Furthermore, pericyte migration may be a secondary effect to the vascular dysfunction or leak. However, further investigation into these intriguing observations is required to understand the events and changes and their implication to vessel stability. Thus, together the results suggest that pericyte alterations together with a loss of the endothelial AJ protein, VE-cadherin, constitute early changes that can be visualized in the vasculature even before plaque formation. These changes are associated with areas of leak and with areas of senescence.

## Discussion

In this study, we have established in AD mouse models that there is breakdown in the integrity of the BBB early in AD development and before the formation of definitive amyloid plaques. The breakdown was assessed at a functional level, with an increase in both para- and transcellular permeability in the cortical microvasculature and molecularly with a widespread decrease in the AJ protein VE-cadherin and in claudin-5 at the sites of leak. In addition, there was enhanced pericyte migration associated with the area of leak, further indicative of an alteration in vascular integrity. As AD pathology develops, the leak size was found to increase and became evident in large vessels. Furthermore, we show that the presence of senescent cells is associated with such leaks, being in the vicinity of sites of leak early in AD while directly associated with leaks as plaques become visible.

There is increasing evidence that BBB dysfunction is an early pathological event in AD as well as in other types of neurological diseases, such as Parkinson’s disease [17, 77]. Our significant finding herein is that the breakdown of the BBB is seen prior to the detection of overt plaques (Fig. 5A, F, and G). Our finding was supported by an early reduction in the expression of the endothelial cell specific adhesion molecule, VE-cadherin, and particularly in the vessels with leak (Fig. 5D & Fig. s10A). VE-cadherin is a known key determinant of microvessel integrity and paracellular permeability [78]. It is shed into the circulation during inflammatory conditions [79, 80] and soluble VE-cadherin is increased in sepsis [81] and atherosclerosis [82]. These diseases show a perturbed endothelium and suggest that detection of plasma VE-cadherin may serve as an early biomarker for BBB dysfunction in AD. Interestingly, claudin-5 expression did not show significant reduction in the cortical vessels at the pre-plaque stage, although there was a decrease in its expression at sites of albumin leak (Fig. 5A). This is in agreement with other studies showing similar temporal changes in claudin-5 expression in 2-month-old and 9-month-old APP/PS1 mice [83].

Since senescence promotes permeability of ECs in vitro (Fig. s1C&D), we investigated areas of leak to determine whether senescent ECs were present in vivo. Surprisingly, we found that there were no vascular SGP^+^ senescent cells (ECs or pericytes) present at the pre-plaque stage of AD. However, there were SGP^+^ senescent cells in close proximity to the areas of leak (Fig. 5E and G, white arrowheads). We have not been able to conclusively subtype these SGP^+^ senescent cells, but their closeness to the vasculature would suggest that they may be astrocytes, perivascular macrophages, or neurons. Further examination with other senescence markers showed high expression of p21 in specific ECs and pericytes (Fig. s8). However, we did not observe any linear association of these cells with vascular leak. Similarly, we observed high p16 expression in specific ECs and pericytes (Fig. s9) but could not determine whether these cells localized with vascular leak. Furthermore, a caveat of this p16-3MR model is while it can detect p16 expression through mRFP, it does not show whether p16 is active in the nucleus as mRFP expression is found predominantly in the cytoplasm [51]. The lack of direct association of these senescent cells with sites of leak although in close vicinity may suggest the influence of the SASP to perturb the BBB. Indeed, one of the major factors secreted by activated macrophages is vascular endothelial cell growth factor (VEGF) also known as vascular permeability factor (VPF) [84] and interestingly VEGF is known to increase the internalization of VE-cadherin [85].

As AD progresses, the changes in BBB integrity could at least partially be attributed to senescent EC and pericytes, as these cells were identified at the site of albumin leak at the time of plaque formation (Fig. 3). Although we estimate only ~ 1% frequency of EC senescence (as judged by SGP positivity) at this stage, their impact on the structure and function of the vessel may be profound, both through their SASP and their alteration of cell–cell contact. In addition, with age, we see a significant increase in senescent EC in mice up to 5% (~ 18–24 months) and in all regions of the brain (data not shown). Gene profiling of microvessels isolated from post-mortem AD patients and transgenic mouse models have also shown vascular senescence in association with tau pathology [86, 87] and tau models exhibit increased vascular permeability. Further studies are required to determine the spatial–temporal senescence of the BBB components in relation to tau pathology.

In finer analysis of the perturbed barrier at the pre-plaque stage, we found pericytes migrating in the vicinity of the vascular leak in between microvessel (Fig. 6Bi, white arrows) but also into areas with no vascular leak. This migration is abnormal as pericytes are largely sessile cells supporting the mature vasculature. However, they can be triggered to proliferate and migrate in response to inflammatory cues or stress [88]. Indeed, pericyte dysfunction has been reported to occur in the early stages of AD [5, 19, 89] and also in the retina of AD [90], a possible window to AD development. Furthermore, amyloid-β oligomers can induce pericyte constriction of human capillaries and this was similarly observed in rat brain slices [91]. Furthermore, APOE4 expression results in activation of pericytes through a cyclophilin A-dependent pathway and with vascular uptake of neurotoxic proteins, and cerebral blood flow reductions that precede neuronal dysfunction [10]. The Angiopoietin-Tie receptor system has been shown to be important for pericyte recruitment and vessel stability [92, 93]. ECs express Tie receptors that bind Angiopoietin-1 expressed by pericytes. Interestingly, our scRNA sequencing data from 8-month-old APP/PS1 (Fig. 4) showed that Tie2 (*Tek*) gene expression was significantly downregulated in ECs. Our results are also in line with human scRNA sequencing showing a decrease in *Tek* expression across arteriole, capillaries, and venous ECs from AD groups compared to controls [94]. The Ang-Tie system could be regulated through the loss of VE-cadherin since stable junctions recruit Tie2-Ang1 into the junctions [95]. Alternately, the Ang-Tie system may itself be a direct early target of oxidative stress induced by the soluble amyloid accumulation, and its decrease may promote pericyte migration and decrease vessel stability.

Pericyte migration was detected in the vicinity of albumin leaks in the older APP/PS1 mice (Fig. 6Bii) and VE-cadherin and claudin-5 were significantly reduced and/or disorganized in these areas of the vasculature (Fig. 3A&B). Interestingly, our preliminary studies would suggest that these migrating pericytes are not senescent (data not shown). Our results are consistent with previous reports describing BBB breakdown [77, 96, 97] and decreases in claudin-5 expression in human AD tissues and amyloidogenic models after significant plaque formation [56, 98]. These findings are also reflected in our scRNA sequencing data where VE-cadherin and claudin-5 expression were significantly downregulated in arteriole, capillary, and venous ECs isolated from the same aged APP/PS1 mice (Fig. 4F). Our scRNA data showed a number of gene sets that indicate the BBB function and integrity were significantly dysregulated (e.g., CLDN5, TEK, ID1, CDH5). Of interest is the decrease in expression of the BBB-specific gene, Mfsd2a. Studies have shown a direct correlation between pericyte coverage and Mfsd2a expression in ECs [36]. Further Mfsd2a reduction promotes caveolae-mediated transcytosis [99] and increased levels of caveolin-1 have been observed in the frontal cortex and hippocampus of post-mortem AD compared to aged-matched control subjects [100]. Caveolin-1 might at least partially regulate some endothelial junctional proteins (e.g., VE-cadherin) through controlling their degradation by MMP9 [101]. These pathways are worthy of further investigation in the development of AD pathology.

Our results in both the APP/PS1 and the APP/PS1 mouse strains confirm that elevated levels of soluble Aβ mediate early detrimental effects on the BBB. The BBB breakdown in turn can lead to accumulation of brain amyloid due to reduced amyloid clearance [102]. Furthermore, with age, there is a change in the uptake of endogenous plasma proteins, and a shift from a predominantly ligand-specific receptor-mediated to a non-specific caveolae transcytosis mechanisms of transport [103] further highlighting an alteration of the BBB function. Thus, it is possible that age-related damage to the BBB results in increases in Aβ burden and accumulation of senescent BBB components adding to the breakdown of the BBB and its associated cognitive decline in sporadic AD. Targeting of these senescent cells as an early therapeutic may have benefit. Such targeting could be through the use of senolytics, drugs that selectively eliminate senescent cells or senomorphics, and drugs that dampen or alter the SASP. Furthermore, our study highlights the possibility that restoring the TJs and AJs to mend the damaged vessels and inhibit vascular leak could also represent a novel therapeutic paradigm in the treatment of AD.

### Supplementary information

Below is the link to the electronic supplementary material. Supplementary figures and their legends need to be inserted here.

Supplementary file1 (DOCX 16 KB)

Supplementary file2 (PDF 70.5 MB)

Supplementary file3 (DOCX 18.1 KB)

## Data Availability

Single cell RNA sequencing data has been successfully uploaded onto GEO. GEO accession number GSE243576.

## Abbreviations

AD: Alzheimer’s disease
Aβ: Amyloid beta
AJs: Adherens junctions
APP: Amyloid precursor protein
Ang: Angiopoietin
APOE4: Apolipoprotein E4
BBB: Blood-brain barrier
BSA: Bovine serum albumin
BF: Brightfield
CapECs: Capillary ECs
CDH5: Cadherin-5
CLDN5: Claudin-5
CNS: Central nervous system
DAPI: 4’, 6-Diamidino-2-phenylindole
ECs: Endothelial cells
FBS: Fetal bovine serum
FITC: Fluorescein isothiocyanate
HCMEC/D3: Human cerebral microvascular endothelial cells
HUVECs: Human umbilical vein endothelial cells
H_2_O_2_: Hydrogen peroxide
ID1: Inhibitor of DNA binding-1
LUC: Renilla luciferase
Mfsd2a: Major facilitator superfamily domain containing 2A
MMP9: Matrix metallopeptidase-9
mRFP: Monomeric red fluorescent protein
OCT: Optimal cutting temperature compound
PFA: Paraformaldehyde
PBS: Phosphate buffered saline
PDGFRβ: Platelet-derived growth factor receptor β
PS1: Presenilin-1
PC: Principal component
P16-3MR: P16-trimodality reporter
SASP: Senescence associated secretory phenotype
SA-β-gal: Senescent β-galactosidase enzyme
SGP: Senescence Green Probe
scRNA-seq: Single-cell RNA sequencing
TEK: Tie2 or Tyrosine-Protein Kinase receptor
TJs: Tight junctions
WT: Wildtype
ZO-1: Zonula occludens-1
