# Supplementary material for: Vascular senescence and leak are features of the early breakdown of the blood–brain barrier in Alzheimer’s disease models
Source: GeroScience. 2023 Oct 2;45(6):3307–31. doi: 10.1007/s11357-023-00927-x (PMC10643714; doi:10.1007/s11357-023-00927-x)
Supplement: Supplementary file 1 — (DOCX 16 KB) [file 11357_2023_927_MOESM1_ESM.docx]

Tab. s1

| **#** | **Senescence signature genes** | **#** | **Senescence signature genes** |
| --- | --- | --- | --- |
| 1 | Hspb1 | 41 | Rdx |
| 2 | H2-D1 | 42 | Ndrg1 |
| 3 | Id1 | 43 | Tek |
| 4 | Bsg | 44 | Pecam1 |
| 5 | Sptbn1 | 45 | Parvb |
| 6 | Plec | 46 | Arhgdia |
| 7 | H2-T23 | 47 | Ndfip1 |
| 8 | Hsp90ab1 | 48 | Rps2 |
| 9 | Igfbp7 | 49 | Rtn4 |
| 10 | Rras | 50 | Cdh5 |
| 11 | Ctnna1 | 51 | Lims2 |
| 12 | Cldn5 | 52 | Pfn1 |
| 13 | Rhoa | 53 | Eef2 |
| 14 | Calr | 54 | Anxa2 |
| 15 | Fxyd5 | 55 | Park7 |
| 16 | Eng | 56 | Eif4h |
| 17 | Nrxn1 | 57 | Rac1 |
| 18 | Sptan1 | 58 | Emp2 |
| 19 | Tln1 | 59 | Rpl14 |
| 20 | Rps3 | 60 | Cd34 |
| 21 | Tgfb2 | 61 | Rack1 |
| 22 | Egfl7 | 62 | Myh9 |
| 23 | Dab2ip | 63 | Sparcl1 |
| 24 | Rpsa | 64 | Iqgap1 |
| 25 | Podxl | 65 | Isg15 |
| 26 | Mprip | 66 | Fbln5 |
| 27 | Utrn | 67 | Cyp1b1 |
| 28 | Macf1 | 68 | Reck |
| 29 | Spon1 | 69 | Vwf |
| 30 | Cpe | 70 | Ehd1 |
| 31 | Cd63 | 71 | Ehd4 |
| 32 | Mdk | 72 | Clic1 |
| 33 | Ptprb | 73 | Adam15 |
| 34 | Ahnak | 74 | Icam2 |
| 35 | Asap1 | 75 | Cd9 |
| 36 | Actb | 76 | Rgcc |
| 37 | Eif5 | 77 | Slc9a3r2 |
| 38 | Cd81 | 78 | Mcam |
| 39 | Gnas | 79 | Cald1 |
| 40 | Mrtfb | 80 | Prnp |
