# Supplementary material for: Vascular senescence and leak are features of the early breakdown of the blood–brain barrier in Alzheimer’s disease models
Source: GeroScience. 2023 Oct 2;45(6):3307–31. doi: 10.1007/s11357-023-00927-x (PMC10643714; doi:10.1007/s11357-023-00927-x)
Supplement: Supplementary file 2 — (PDF 70.5 MB) [file 11357_2023_927_MOESM2_ESM.pdf]

Fig. s1

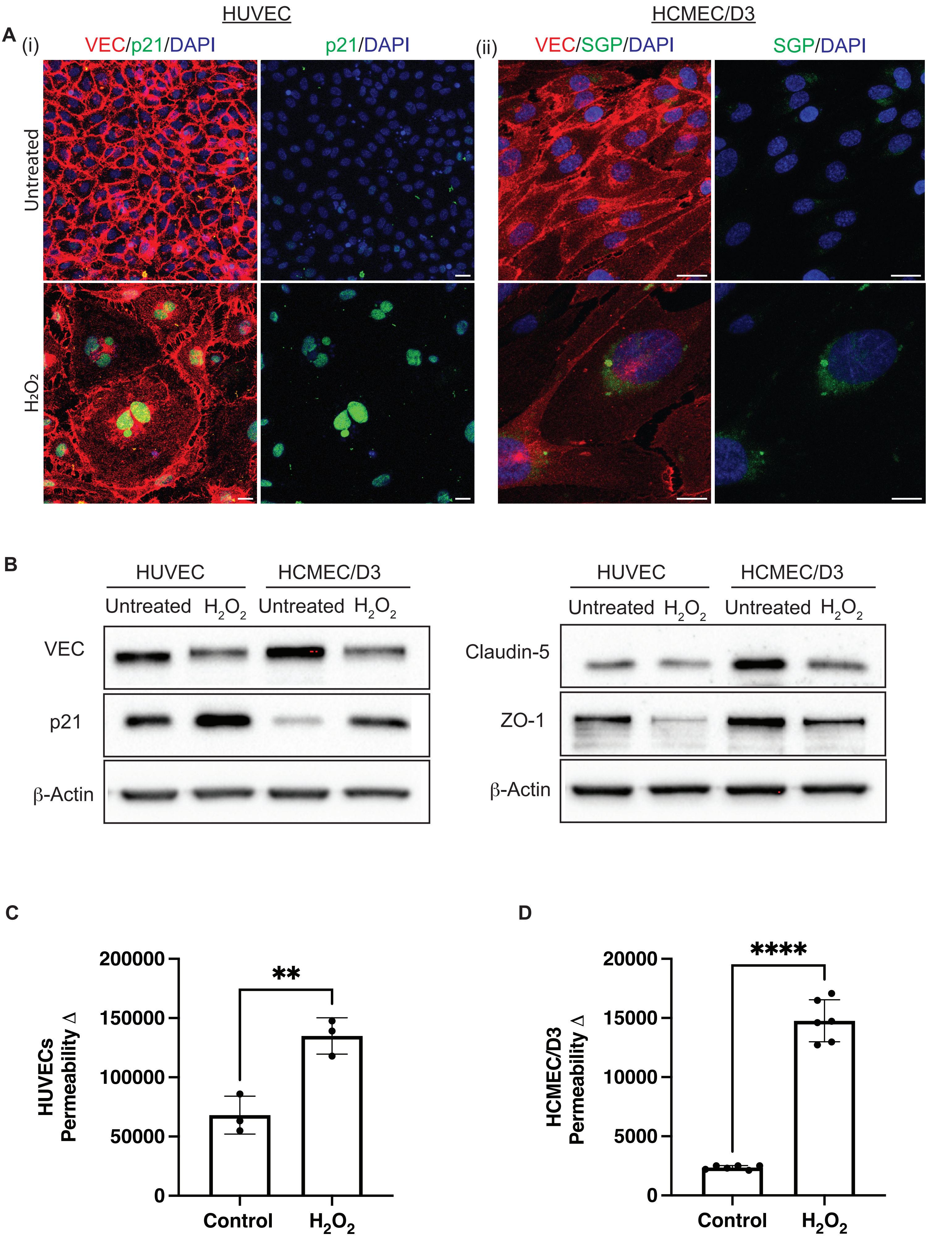
HUVEC
HCMEC/D3
A
(i)
VEC/p21/DAPI
p21/DAPI
(ii)
VEC/SGP/DAPI
SGP/DAPI
Untreated
H2O2
B
HUVEC
HCMEC/D3
Untreated
H2O2
Untreated
H2O2
VEC
p21
β-Actin
HUVEC
HCMEC/D3
Untreated
H2O2
Untreated
H2O2
Claudin-5
ZO-1
β-Actin
C
200000
150000
100000
50000
0
HUVECs Permeability Δ
**
Control
H2O2
D
20000
15000
10000
5000
0
HCMEC/D3 Permeability Δ
****
Control
H2O2

Fig. s2

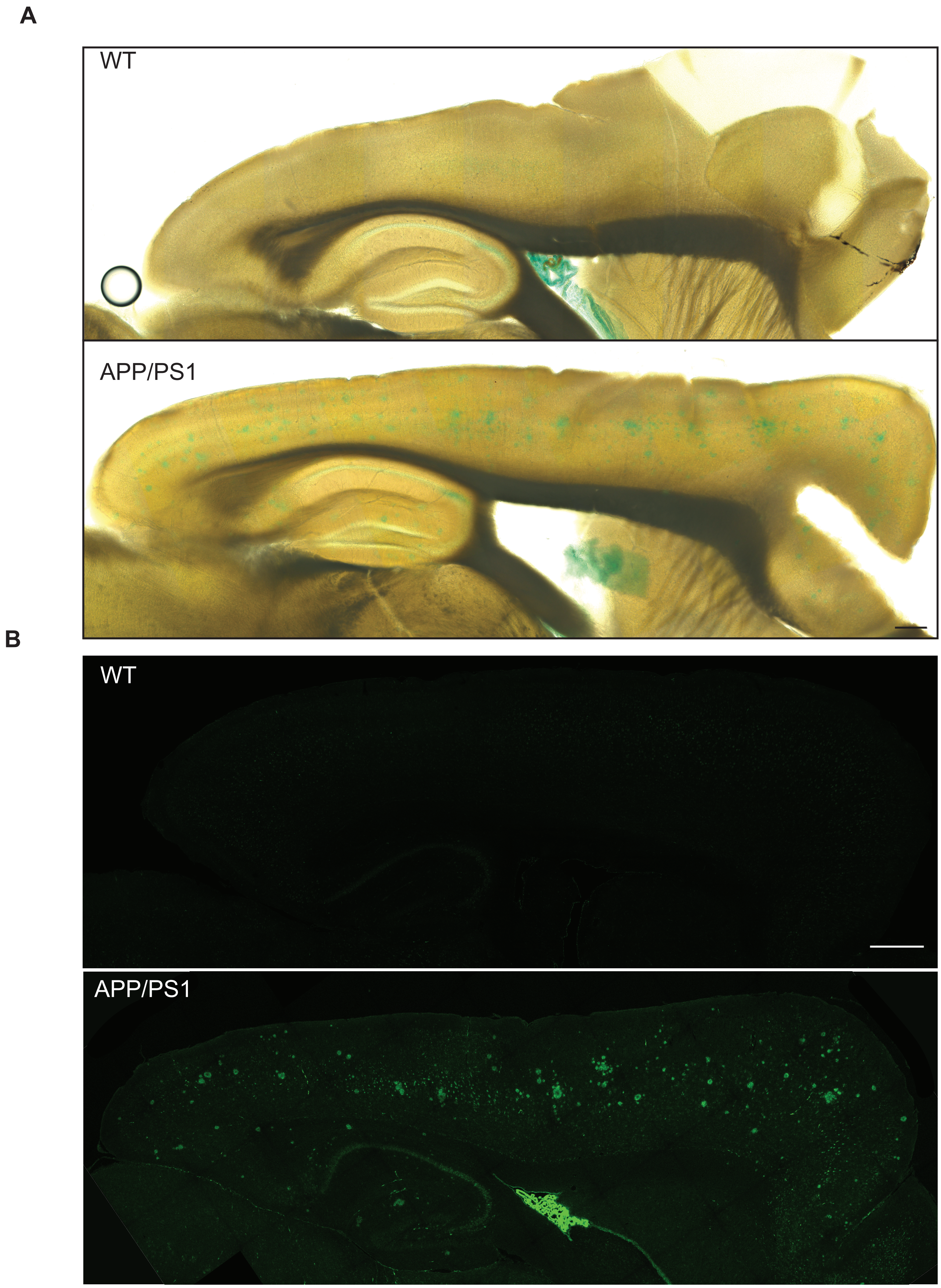

Fig. s3

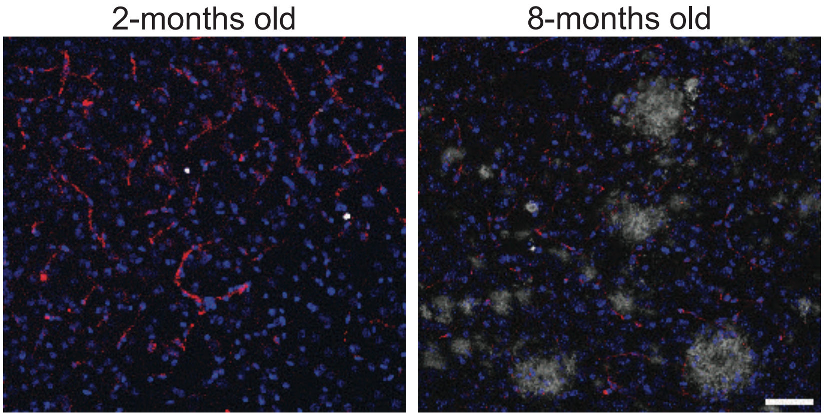

Fig. s4

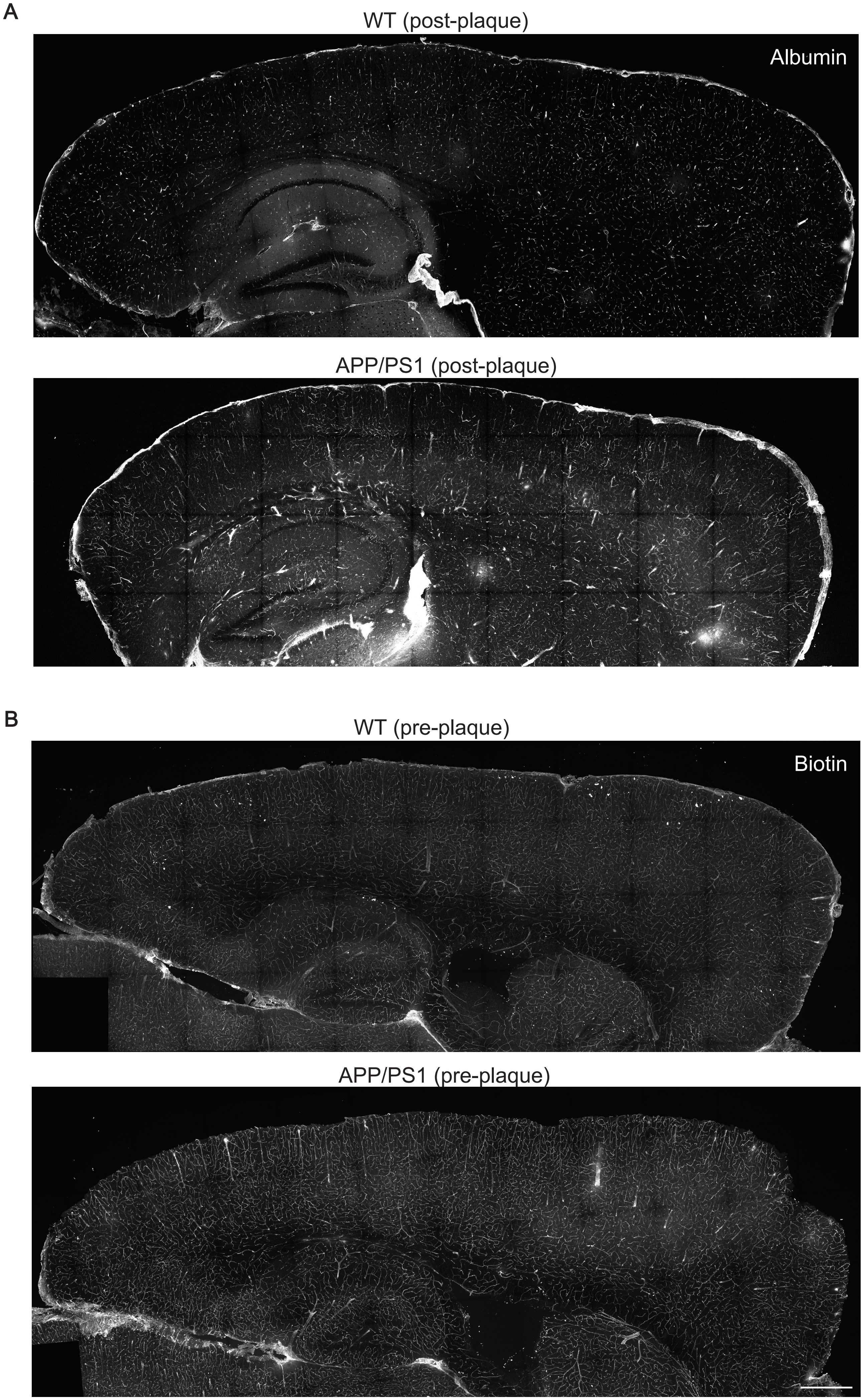
A
WT (post-plaque)
Albumin
APP/PS1 (post-plaque)
B
WT (pre-plaque)
Biotin
APP/PS1 (pre-plaque)

Fig. s5

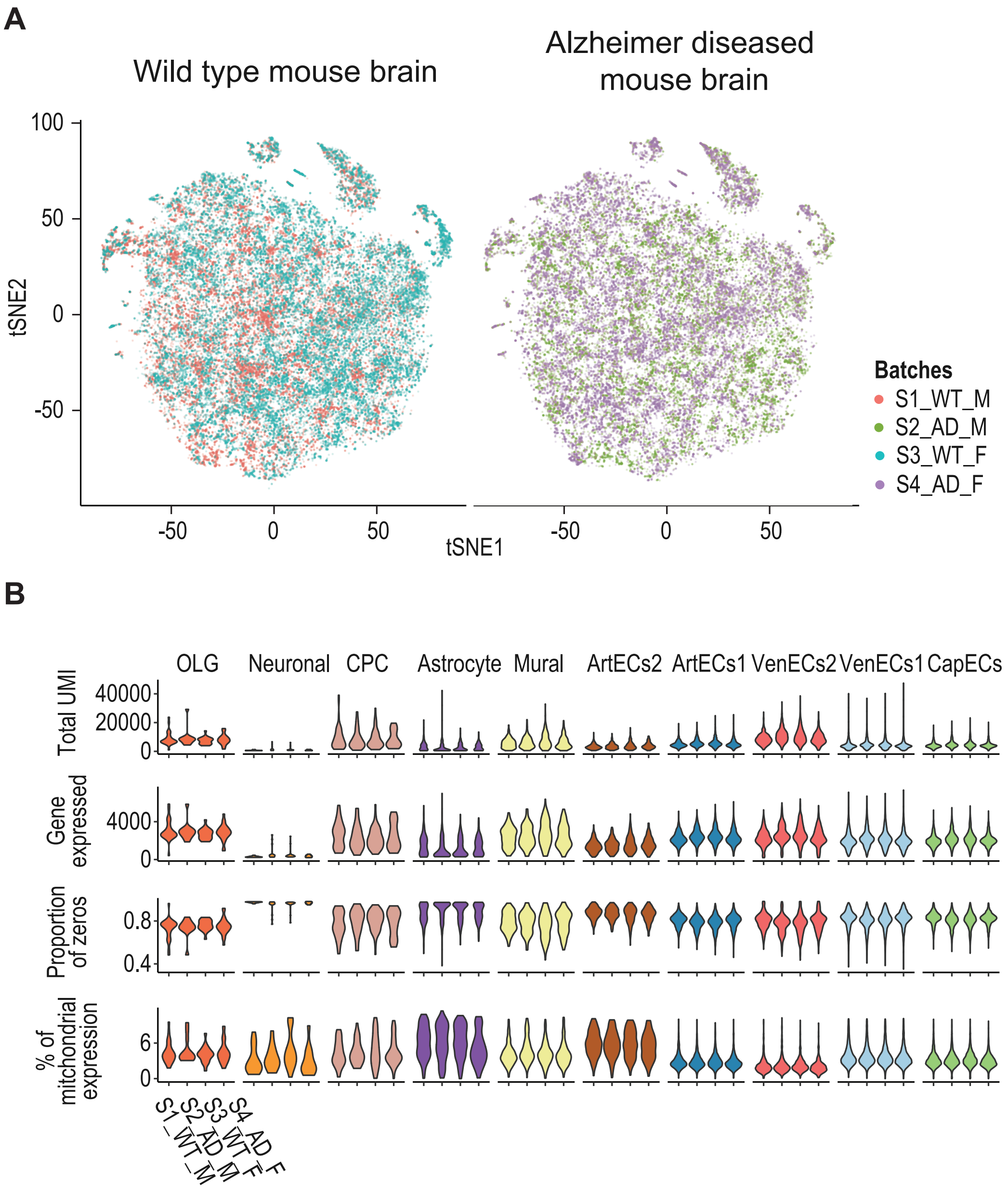
A
Wild type mouse brain
Alzheimer diseased mouse brain
tSNE2
tSNE1
Batches
S1_WT_M
S2_AD_M
S3_WT_F
S4_AD_F
B
OLG
Neuronal
CPC
Astrocyte
Mural
ArtECs2
ArtECs1
VenECs2
VenECs1
CapECs
Total UMI
Gene expressed
Proportion of zeros
% of mitchondrial expression

Fig. s6

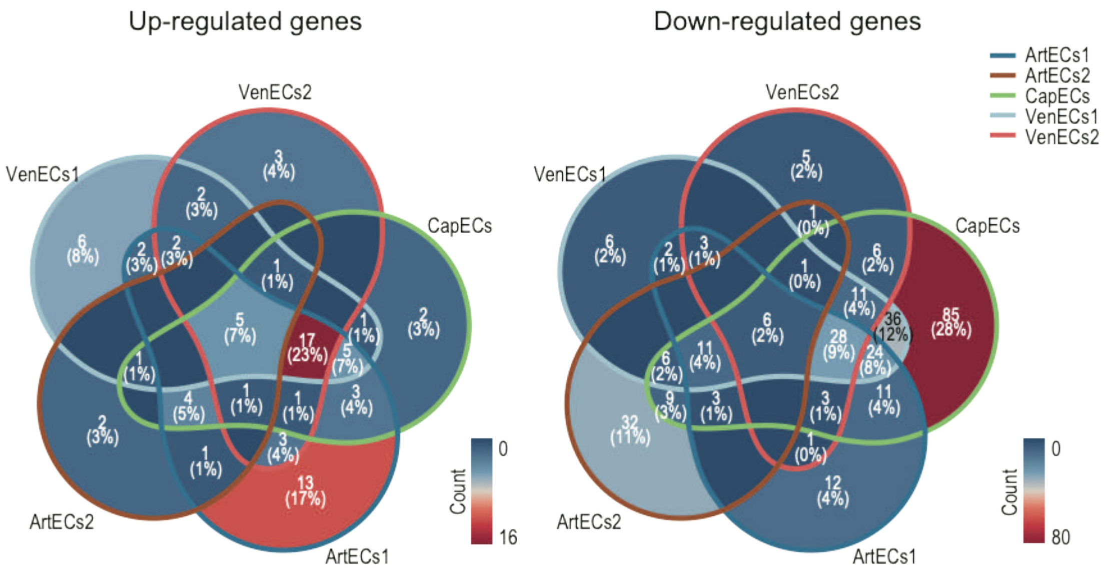
Up-regulated genes
Down-regulated genes
VenECs2
VenECs1
CapECs
ArtECs2
ArtECs1
Count
0
16
80
ArtECs1
ArtECs2
CapECs
VenECs1
VenECs2

Fig. s7

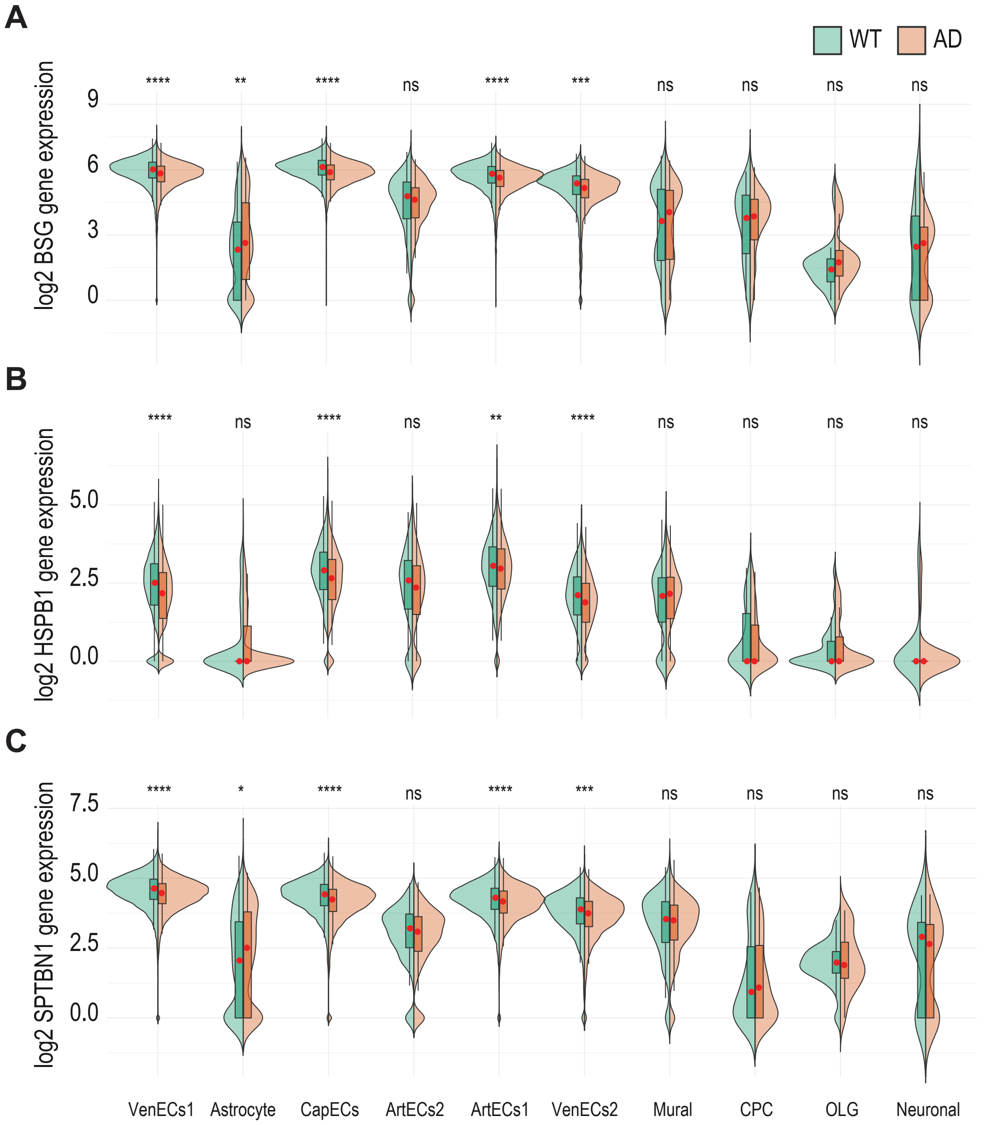
A
B
C
WT
AD
log2 BSG gene expression
log2 HSPB1 gene expression
log2 SPTBN1 gene expression
VenECs1
Astrocyte
CapECs
ArtECs2
ArtECs1
VenECs2
Mural
CPC
OLG
Neuronal

Fig. s8

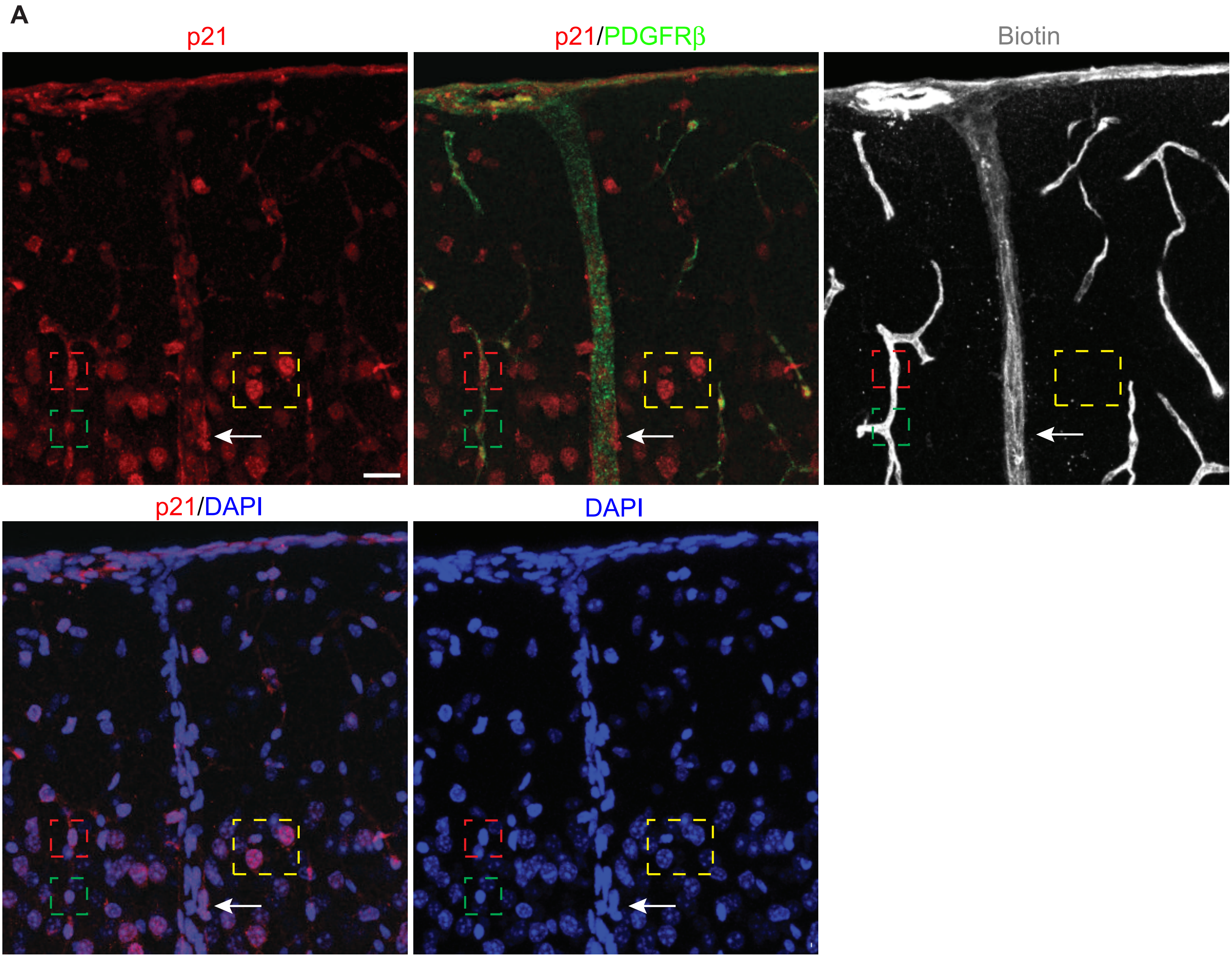
A
p21
p21/PDGFRβ
Biotin
p21/DAPI
DAPI

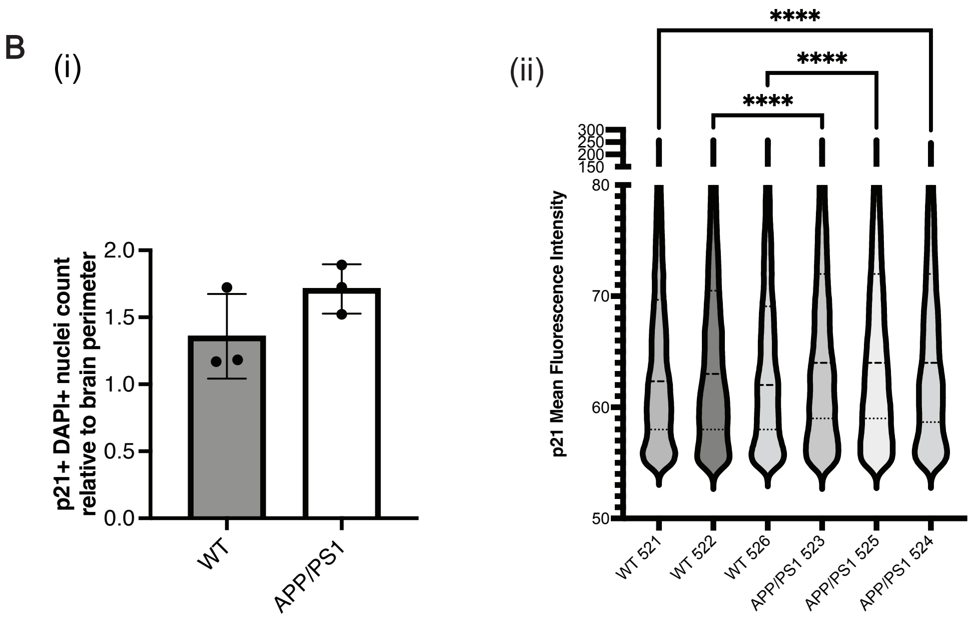
B
(i)
p21+ DAPI+ nuclei count relative to brain perimeter
2.0
1.5
1.0
0.5
0.0
WT
APP/PS1
(ii)
****
****
****
p21 Mean Fluorescence Intensity
300
250
200
150
80
70
60
50
WT 521
WT 522
WT 526
APP/PS1 523
APP/PS1 525
APP/PS1 524

Fig. s9

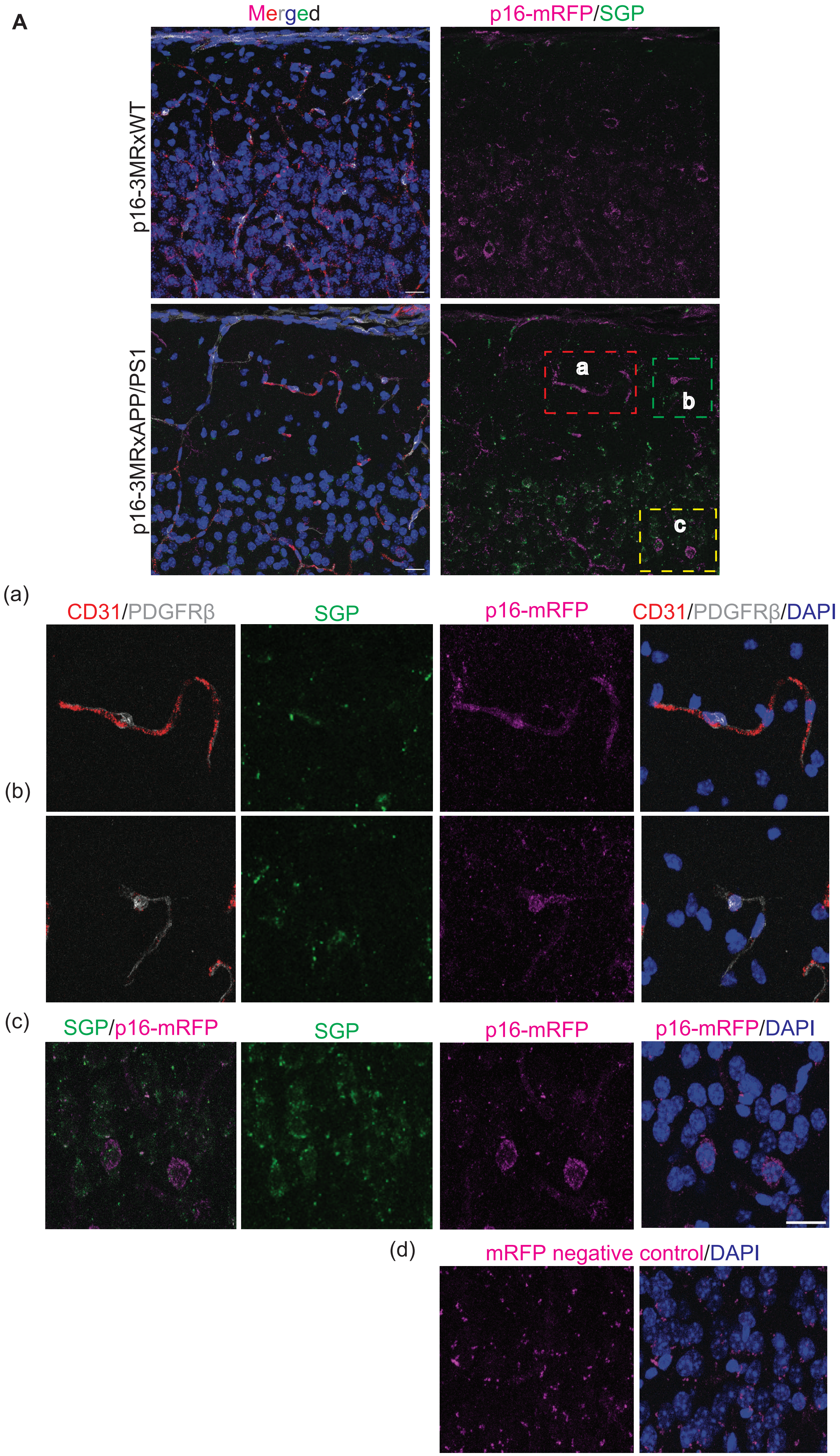

A
Merged
p16-mRFP/SGP
p16-3MRxWT
p16-3MRxAPP/PS1
a
b
c
(a)
CD31/PDGFRβ
SGP
p16-mRFP
CD31/PDGFRβ/DAPI
(b)
(c)
SGP/p16-mRFP
SGP
p16-mRFP
p16-mRFP/DAPI
(d)
mRFP negative control/DAPI

Fig. s10

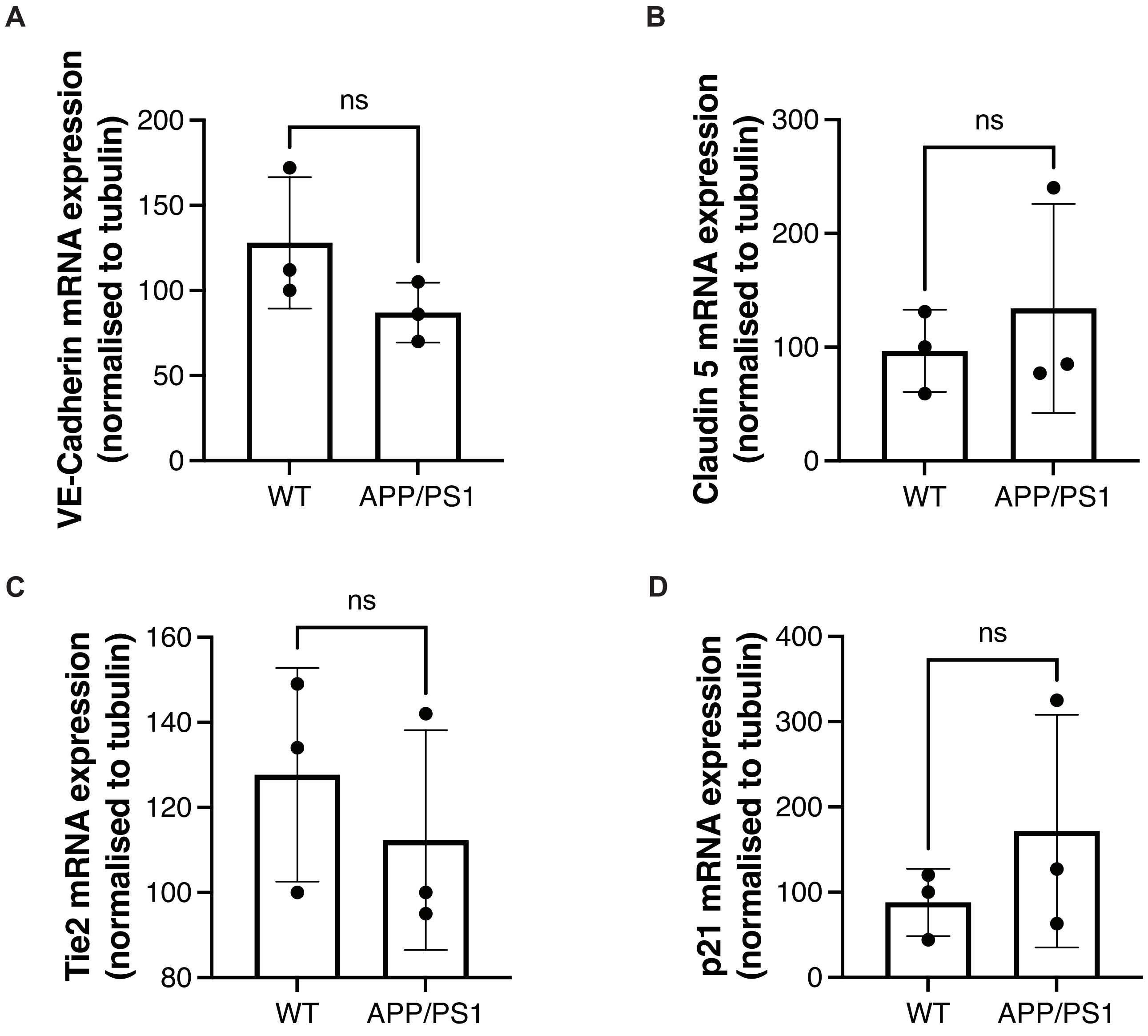
A
VE-Cadherin mRNA expression
(normalised to tubulin)
200
150
100
50
0
ns
WT
APP/PS1
B
Claudin 5 mRNA expression
(normalised to tubulin)
300
200
100
0
ns
WT
APP/PS1
C
Tie2 mRNA expression
(normalised to tubulin)
160
140
120
100
80
ns
WT
APP/PS1
D
p21 mRNA expression
(normalised to tubulin)
400
300
200
100
0
ns
WT
APP/PS1

Fig. s11

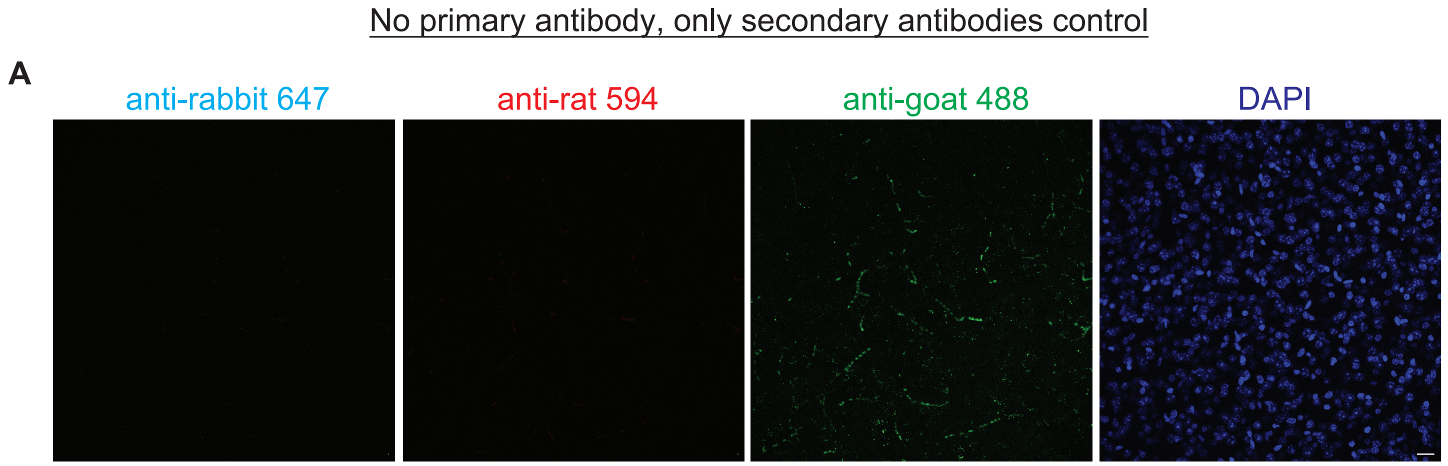
No primary antibody, only secondary antibodies control
A
anti-rabbit 647
anti-rat 594
anti-goat 488
DAPI

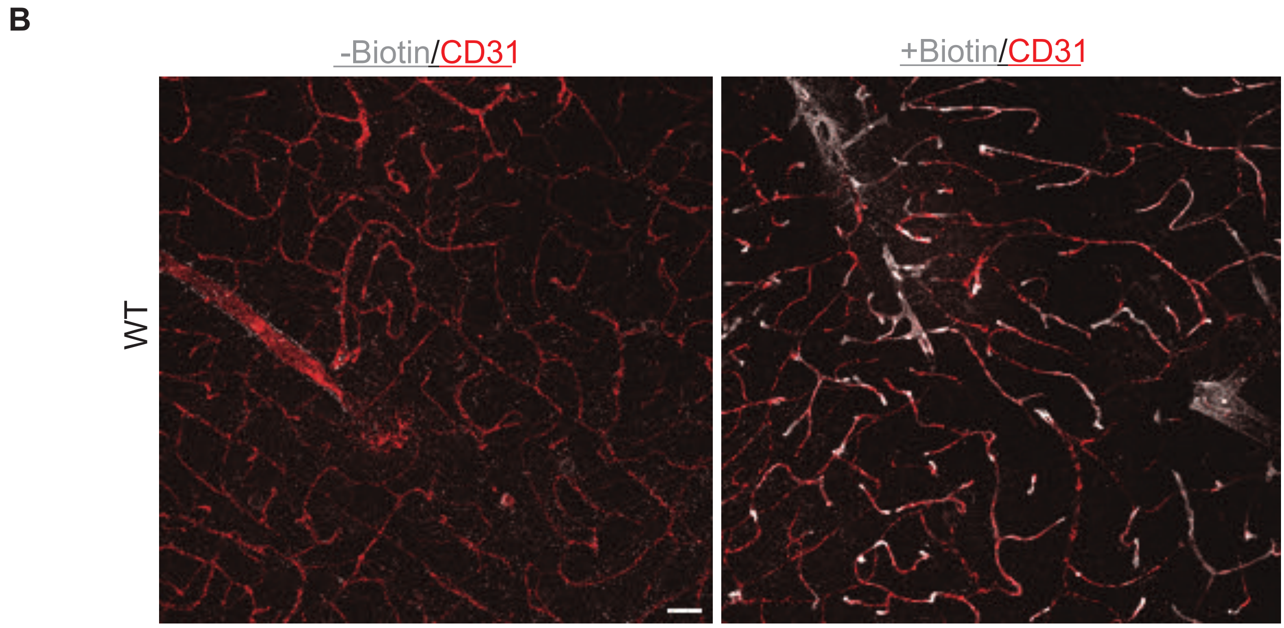
B
-Biotin/CD31
+Biotin/CD31
WT
