# Supplementary material for: Vascular senescence and leak are features of the early breakdown of the blood–brain barrier in Alzheimer’s disease models
Source: GeroScience. 2023 Oct 2;45(6):3307–31. doi: 10.1007/s11357-023-00927-x (PMC10643714; doi:10.1007/s11357-023-00927-x)
Supplement: Supplementary file 3 — (DOCX 18.1 KB) [file 11357_2023_927_MOESM3_ESM.docx]

**Supplementary Figures**

**Fig. s1 Senescence induction using H_2_O_2_ in primary HUVECs and in HCMEC/D3 cell line.**

**(Ai-ii)** Untreated HUVECs and HCMEC/D3 cells (top row) show strong and continuous expression of VE-cadherin (VEC, red) compared to H_2_O_2_-treated cells (bottom row). **(Ai)** Large senescent HUVEC cells also have increased expression of p21 (green) localised to the nucleus (DAPI, blue) compared to untreated controls. **(Aii)** Senescent HCMEC/D3 cells are larger compared to untreated cells and have expression of SGP (green). **(B)** Western blot results show a significant decrease in VE-cadherin, claudin-5 and ZO-1 and increase in p21 expression in H_2_O_2_-treated group compared to untreated controls, respectively. **(C)** Permeability assay shows the increase in FITC-dextran dye permeability in H_2_O_2_-treated HUVECs in comparison to untreated controls. **(D)** H_2_O_2_-treated HCMEC/D3 cells have increased permeability compared to untreated controls.

Western blot data is from 4-6 different HUVEC lines. Permeability assay data is from 3 different HUVEC lines and 6 replicates of the HCMEC/D3 cell line represented as mean ± standard deviation. Unpaired t-test was used to determine permeability between untreated and treated cells. ns: p-value > 0.05; *p-value ≤ 0.05; **p-value ≤ 0.01; ***p-value≤ 0.001****: p-value ≤ 0.0001. Scale bar for **Fig. Ai-ii** = 20 µm.

**Fig. s2 Comparison between conventional SA-β-gal and SGP stain.**

**(A)** This figure shows the typical blue stain produced by the conventional SA-β-gal in sagittal brain section from a 6-month old APP/PS1 mouse. **(B)** Strong expression of SGP stain (green) is found in sagittal brain section from an 8-month old APP/PS1 mouse. APP/PS1 mice typically exhibit amyloid plaques from as early as 3 to 4 months. There is little to no staining of SA-β-gal and SGP found in the wildtype mouse. The conventional SA-β-gal and SGP display similar patterns of expression at a macroscopic level, where both stains accumulate in the amyloid plaques. Scale bar= 500 µm.

**Fig. s3 Amyloid plaque formation in APP/PS1 mice at 2-months and 8-months of age.**

Representative image of APP/PS1 mouse stained with pan-amyloid marker (grey) and blood vessels (red) at 2-months, before plaques are formed and at 8-months, after significant plaque formation. Scale bar= 50 µm

**Fig. s4 Vascular leak of endogenous albumin and biotin at different stages of amyloid plaque formation.**

**(A)** Leakage of endogenous albumin (white) was undetected in wildtype mice (top). Significant albumin leak was detected in APP/PS1 mice after plaque formation (bottom). **(B)** Biotin leak was not detected in wildtype mice, as the injected dye was localized to the vasculature (top). Biotin dye (white) was detected within the brain parenchyma of APP/PS1 mice before plaque formation, which is an indication of vascular leak in these mice. Scale bar= 500 µm.

**Fig. s5 Quality control of the mouse brain scRNA-seq dataset. (A)** tSNE representation of the transcriptomes of single cells. Cells are coloured by batch in healthy (left) and diseased brain (right). **(B)** Number of reads (unique molecular identifier; UMI), genes quantified, proportion of zeros, and percentage of mitochondrial genes expressed in cells for each cell type and batch.

**Fig. s6 Overlap of differentially regulated genes between the endothelial cell types.** Venn diagram illustrating the number of genes overlapping among the significantly up-regulated (left) and down-regulated (right) genes (FDR-adjusted p-value < 0.05 and absolute log2 fold-change > 0.1). The total percentage of overlap is denoted within brackets for intersections greater than 0% of overlaps. The colour scale denotes the absolute number of genes in each gene set.

**Fig. s7 Gene expression of example genes related to adhesion and the blood brain barrier.** The normalized gene expression of **(A)** BSG, **(B)** HSPB1, and **(C)** SPTBN1 in healthy (WT, green) and Alzheimer’s disease (AD, red) brain cells is visualized as split violin and box plots for each cell type. The median is denoted as red circles. The box denotes the inter-quartile range. Outliers have been omitted to facilitate visualization. The student’s *t*-test was used to compare the difference in mean gene expression between healthy and diseased cells. ns: p-value > 0.05; *p-value ≤ 0.05; **p-value ≤ 0.01; ***p-value≤ 0.001****: p-value ≤ 0.0001.

**Fig. s8 Nucleus associated p21 expression in APP/PS1 mice before plaque formation.**

**(A)** Endothelial cell (red box), pericytes (green box) and neurons (yellow box) have been identified to express high levels of p21. Perivascular cells also express p21 as shown by the white arrows. Biotin (white) leak was not observed. Co-staining of p21 and DAPI (blue) shows p21 to be associated with the nucleus. **(Bi)** Measurement of the number of nuclei positive for p21 was found to be increased in APP/PS1 mice compared to WT. **(Bii)** Violin plot of the mean fluorescence intensity in cortical sections of the brain showed an increase in p21 expression in APP/PS1 mice compared to WT littermates. Scale bar= 20 µm. The student’s t-test was used to compare the difference in number of p21^+^ nuclei between WT and APP/PS1 mice. Ordinary one-way ANOVA was used for comparisons of cortical p21 expression between WT and APP/PS1 mice. Data is from n= 3 mice per group and represented as mean ± standard deviation. ns: p-value >0.05, *p-value ≤ 0.05; **p-value ≤ 0.01; ***p-value≤ 0.001****: p-value ≤ 0.0001.

**Fig. s9 P16 expression in p16-3MRxAPP/PS1 and p16-3MRxWT mice at the pre-plaque formation stage.**

**(A)** Representative images of p16-mRFP (magenta), CD31 (red), SGP (green) PDGFRβ (white) and DAPI (blue) in the cortical region in the crossbred mice. Co-staining of p16-mRFP and SGP showed there is minimal SGP expression in WT mice compared to APP/PS1 mice. The majority of the P16-mRFP expression did not colocalize with SGP expression. P16-mRFP expression is primarily detected in the microvasculature (red box; a) pericytes (green box; b) and some neurons (yellow box; c). **(a)** Closer examination shows SGP and p16-mRFP expression was localised to CD31 microvessel. Adjacent pericyte wrapped around this vessel did not express SGP or p16-mRFP. **(b)** An example of a pericyte positive for PDGFRβ and p16-mRFP but lacked SGP expression. **(c)** An example of a pair of neurons determined by their distinctive and large nucleus were positive for p16-mRFP. In contrast, other p16-mRFP negative neurons with similar nuclear pattern have high levels of SGP. **(d)** APP/PS1 mice that do not express trimodal (3MR) reporter was stained for mRFP and showed negative staining for mRFP. Dots observed in this case are non-specific staining. Scale bar= 20 µm.

**Fig. s10 Validation of VE-cadherin and Claudin-5 expression in isolated microvessels at pre-plaque stage.**

Quantitative PCR of endothelial **(A)** *Cdh5* (VE-cadherin), **(B)** *Cldn5* (Claudin-5), **(C)** *Cdkn1a* (p21), and **(D)** *Tek* (Tie2) expression in wildtype (WT) and APP/PS1 littermates. Data is from n= 3 mice per group and represented as mean ± standard deviation. Unpaired t-test was used to determine gene expression between WT and APP/PS1 mice. ns: p-value>0.05; *p-value≤ 0.05.

**Fig. s11 Negative staining controls for antibodies used and biotin leak assays.**

**(A)** Representative images of no primary antibody staining in APP/PS1 transgenic mice brain tissue. Only secondary antibody was used as controls to show specific staining of antibodies used in this study. Fluorophores used was anti-rabbit AlexaFluor 647, anti-rat AlexaFluor 594, anti-goat AlexaFluor 488 and DAPI for nuclei staining. **(B)** WT mouse without biotin injection was used as negative control for biotin leak staining. CD31 staining (red) was used to label the vasculature. WT mouse with biotin injection have positive staining of biotin (white) that localized to the vasculature. Scale bar= 20 µm.
